# The Neural Correlates of Problem States: Testing fMRI Predictions of a Computational Model of Multitasking

**DOI:** 10.1371/journal.pone.0012966

**Published:** 2010-09-23

**Authors:** Jelmer P. Borst, Niels A. Taatgen, Andrea Stocco, Hedderik van Rijn

**Affiliations:** 1 Department of Artificial Intelligence, University of Groningen, Groningen, The Netherlands; 2 Institute for Learning and Brain Sciences, University of Washington, Seattle, Washington, United States of America; 3 Department of Psychology, University of Groningen, Groningen, The Netherlands; The University of Melbourne, Australia

## Abstract

**Background:**

It has been shown that people can only maintain one problem state, or intermediate mental representation, at a time. When more than one problem state is required, for example in multitasking, performance decreases considerably. This effect has been explained in terms of a problem state bottleneck.

**Methodology:**

In the current study we use the complimentary methodologies of computational cognitive modeling and neuroimaging to investigate the neural correlates of this problem state bottleneck. In particular, an existing computational cognitive model was used to generate *a priori* fMRI predictions for a multitasking experiment in which the problem state bottleneck plays a major role. Hemodynamic responses were predicted for five brain regions, corresponding to five cognitive resources in the model. Most importantly, we predicted the intraparietal sulcus to show a strong effect of the problem state manipulations.

**Conclusions:**

Some of the predictions were confirmed by a subsequent fMRI experiment, while others were not matched by the data. The experiment supported the hypothesis that the problem state bottleneck is a plausible cause of the interference in the experiment and that it could be located in the intraparietal sulcus.

## Introduction

One of the challenges for research on multitasking is to explain why some tasks can be performed together without a problem (e.g., talking and walking), while other tasks clearly interfere with each other (e.g., talking and reading). According to so-called multiple-resource theories, interference occurs when multiple tasks require the same cognitive or peripheral resources (e.g., [1]–[3]). An obvious example is our visual system: we can only look at one thing at a time. There is empirical evidence that the same principle might hold for cognitive resources: for instance indicating that we can only retrieve one fact at a time from declarative memory (e.g., [4]). The impact of a concurrent request to a particular resource depends on the time scale of multitasking: whether it is truly concurrent multitasking (e.g., driving and calling), or whether the task can be characterized as ‘sequential multitasking’ (e.g., writing a paper and answering the phone; [5]).

A resource that causes considerable interference in both concurrent and sequential multitasking is the problem state resource. This resource is used for maintaining intermediate task representations. For instance, when mentally solving the algebra problem 3*x*−10 = 2 it is used to store 3*x* = 12 (e.g., [6]). In a series of experiments we have shown that the problem state resource acts as a bottleneck in sequential multitasking [7]. When multiple tasks needed to store intermediate results, interference was observed. However, when only one of the tasks required access to intermediate results, no interference was found. To account for these experimental results, we developed a computational cognitive model that showed that a ‘problem state bottleneck’ could explain the behavioral data.

The goal of this paper is to explore the neural underpinnings of the problem state bottleneck and to further validate our cognitive model. To these ends, the model was used to generate *a priori* predictions of hemodynamic activation patterns in five predefined brain areas for a triple-task. Subsequently, an fMRI experiment was conducted, and the model predictions were compared to the data. Some of the predictions were confirmed, while others did not match with the data. In general the results corroborate the model and provide further evidence (see e.g., [6]) that the intraparietal sulcus is a probably location for the problem state resource. In the remainder of this paper we will first introduce the theory related to the problem state bottleneck, followed by a description of the experiment, the model, and the fMRI predictions. Finally, we will discuss the correspondence between the predictions and the fMRI data, and the implications for the problem state bottleneck hypothesis.

### The Problem State Bottleneck

The problem state resource is the part of working memory responsible for storing intermediate representations in a task. For instance, the problem state can be used to store an intermediate state of an algebra problem, as mentioned above. An everyday example is asking for driving directions, during which one needs the problem state resource to store at which street one should turn to arrive at the destination. Note that if the same information is present in the world, that is, if one works out the algebra problem on paper or follows road signs to the destination, it is not necessary to maintain a problem state. An important functional characteristic of the problem state resource is that its contents are directly accessible for the task at hand. This in contrast to other elements in working memory, which are only available at a time cost (e.g., [8]).

The concept of a central problem state resource originates from a series of neuroimaging experiments by Anderson and colleagues, who found that the Blood-Oxygen Level-Dependent (BOLD) signal in the posterior parietal cortex correlates with the number of transformations of mental representations (e.g., [6], [9]–[11]).

Previously, we have conducted a number of experiments investigating the nature of this resource [7], [12]. These experiments show that people can only maintain one problem state at a time. When a problem state was required for more than one task, performance decreased considerably, indicating a processing bottleneck. To account for these results we constructed a cognitive model based on the threaded cognition theory [2] and the cognitive architecture ACT-R [13]. The model fits well to the data (see the next section), further corroborating the hypothesis of a problem state bottleneck as a plausible explanation of multitask interference. The next section will discuss how the model was used to generate fMRI predictions for the current study.

### A Priori Model Predictions

To validate cognitive models, it is common practice to compare model data to behavioral data. For instance, if response times and accuracy scores correspond well between model and data, it is assumed that a model gives a plausible explanation of the data. However, many cognitive models have a complexity that cannot be accounted for by using only behavioral measurements (e.g., [14], [15]). One solution is to use predictions: first use a cognitive model to predict the outcome of an experiment, and only conduct the experiment afterwards ([16]; see for examples [17], [18]). Nevertheless, there are so many degrees of freedom in developing a model that models are often under-constrained by behavioral data. To increase the constraints on models that are developed in the cognitive architecture ACT-R, a methodology was developed for mapping model activity on brain activity (for a concise explanation, see [19]). This way, models are not only constrained by behavioral data, but also by neuroimaging data. The next sections will describe how this methodology was used to generate *a priori* neuroimaging predictions from our model. We will first describe the experimental setup and the model itself, followed by the actual predictions.

#### The triple task

The task for which we generate BOLD-predictions is a triple task in which participants have to perform a subtraction task, a text entry task, and a listening comprehension task (similar to Experiment 3 in [7]). The subtraction and text entry tasks both have an easy version for which maintaining a problem state *is not* required to perform the task, and a hard version for which maintaining a problem state *is* required to perform the task correctly. In half of the trials, participants also had to listen to a short story on which they were quizzed after the trial. To measure baseline performance on the listening task, we included an ‘Only Listening’ condition in which participants only had to do the listening task. Thus, the experiment has a 2×2×2+1 design (Subtraction Difficulty (easy/hard)×Text Entry Difficulty (easy/hard)×Listening (yes/no)+Only Listening).

**Figure 1:**
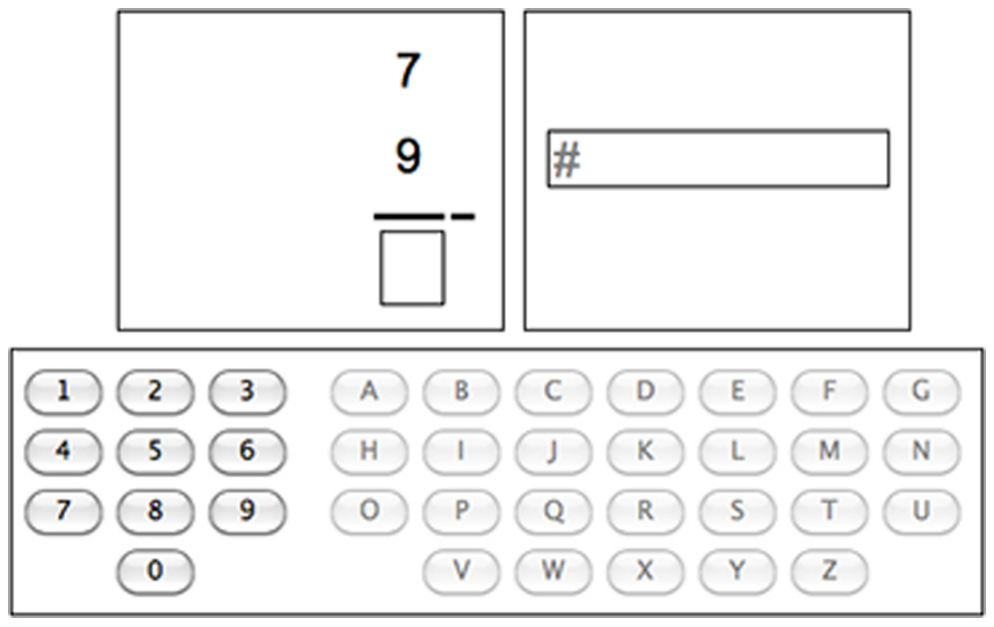
Interface of the experiment. Note that the disabled task is masked by #-marks.

Figure 1 shows the graphical interface of the experiment. The subtraction and text entry tasks were presented at the same time on two different panels of the interface; participants had to alternate between these tasks. After entering a digit in the subtraction task, the subtraction panel was disabled, forcing the participant to subsequently enter a letter. After entering a letter, the text entry panel was disabled and the subtraction panel became available again. In half of the trials, the listening task had to be performed at the same time as the other two tasks. Thus, this paradigm allows us to study both concurrent (listening and subtraction/text entry), and sequential multitasking (alternating between subtraction and text entry).

The interface for the subtraction task is shown in the left panel of Figure 1. In the subtraction task participants had to solve multi-column subtraction problems in standard right-to-left order. However, at each point in time, only one column was visible. Although the problems were presented column by column, the participants were trained to perceive the separate columns in a trial as one 10-column subtraction problem (in the practice phase participants started out with a normal 10-column layout, only later they switched to solving the problems column by column). Participants had to enter the digits by clicking on the on-screen keypad with the mouse. In the easy, no problem state version, the upper digit was always larger or equal to the lower one; these problems could be solved without ‘borrowing’. In contrast, the hard version required participants to borrow six times out of 10 possible columns. The assumption, supported by the results of [7], is that participants have to use their problem state resource to keep track of whether a ‘borrowing’ is in progress.

The interface for the text entry task is shown on the right in Figure 1. Participants had to enter 10-letter strings by clicking on the on-screen keypad. In the easy version these strings were presented one letter at a time and participants had to click the corresponding button on the keypad. In the hard version, a 10-letter word was presented once at the start of a trial. Once a participant clicked on the first letter, the word disappeared and the remaining letters had to be entered one at a time, without feedback. Thus, after the initial presentation of the string in the hard condition, participants could neither see what word they were entering, nor what they had already entered. Results by [7] provide evidence that participants use their problem state resource to keep track of the process.

The listening comprehension task had to be performed during half of the trials. This task consisted of listening to a short story about which a multiple-choice question would be asked at the end of the trial. After answering the question, participants received accuracy feedback. According to existing models of language processing in ACT-R, this task does not require maintenance of problem states, but draws on different cognitive resources [20], [21]. Furthermore, the listening task did not affect the problem state-related outcomes of Experiment 3 in [7], also indicating an absence of problem state usage. This, in turn, indicates that problem state interference does not depend on the number of tasks, but on the particular cognitive resources used by the tasks. In the ‘only listening’ condition a fixation cross was shown instead of the subtraction and text entry tasks.

Because participants had to alternate between the subtraction and text entry tasks after every letter and digit, they had to maintain intermediate state information for the other task (when it was hard) while giving a response on the current task. Based on the threaded cognition theory [2], we predicted that it is not possible to maintain more than one problem state at a time, and therefore expected to find interference when participants have to use a problem state for both tasks. As the listening task was assumed not to use the problem state resource, it was expected that problem state interference was independent of the listening task.

The results of the behavioral experiment of [7] were as follows. Response times were considerably higher and accuracy lower in the hard subtraction – hard text entry condition than in the other conditions. In fact, we found an interaction effect of Subtraction Difficulty and Text Entry Difficulty both in response times and accuracy. The listening task had little behavioral effect; it was limited to a small increase in response times in the subtraction task when the listening task was added. Because the subtraction and text entry tasks were performed sequentially, it is unlikely that the observed interaction was caused by condition-specific differences between the easy and hard conditions: only problem states had to be maintained while doing the other task (see for a much more elaborate discussion of these results [7] in particular Experiment 2). Thus, in line with the problem state bottleneck hypothesis, the strongest interference occurred in the hard subtraction - hard text entry condition, indicating that participants could not maintain two problem states at the same time.

#### The cognitive model

To account for these results, a model was developed in the cognitive architecture ACT-R, using the threaded cognition theory to handle multitasking. First we will introduce ACT-R and threaded cognition, followed by a description of the model itself.

The cognitive architecture ACT-R [13] describes human cognition as a set of independent modules – cognitive resources – that interact through a central production system. For instance, it uses visual and aural modules for perception and a motor module to interact with the world. Besides these peripheral modules ACT-R also has a number of central cognitive modules: the procedural module that implements the central production system, the declarative memory module, the goal module, and the problem state module. All modules operate in parallel, but each module in itself can only proceed serially [22]. Thus, the visual module can only perceive one object at a time and the memory module can only retrieve one fact at a time.

Threaded cognition [2], [5], [23] extends ACT-R by allowing multiple tasks – called threads – to be active at the same time. However, because the cognitive resources are serial in nature, the key assumption of threaded cognition is that although *several tasks* can be active at the same time, a particular *resource* can only be used by a *single task* at a time, and thus acts as a bottleneck when required by multiple tasks concurrently.

Of particular importance for the tasks at hand is ACT-R's problem state module. Although this module can hold a problem state that is accessible at no time cost, changing or restoring a problem state has been estimated to take a relatively long time (a value of 200 ms has provided a good fit in previous ACT-R models, and has been left unchanged in our models; e.g., [24], [25]). Because the problem state module can only hold one chunk of information, the module's contents have to be swapped when multiple problem states are required. When a problem state is replaced, the previous problem state remains available in long-term memory, and it can be recalled when required. However, as both retrieving an old problem state from declarative memory and updating the problem state takes time, using multiple problem states causes considerable interference. An additional effect of swapping problem states is that because older problem states need to be retrieved from memory, it is possible to retrieve an incorrect problem state from memory, resulting in behavioral errors.

The model for the triple task consists of three independent threads, one for the subtraction task, one for the text entry task, and one for the listening task. The subtraction and text entry threads use the visual module to perceive the stimuli and the manual module to operate the mouse. In the easy condition of the subtraction task, the model perceives the digits, retrieves a fact from memory (e.g., 5−2 = 3) and clicks on the corresponding button. The procedure is the same in the hard condition, up to the point when borrowing becomes necessary. When the model retrieves a fact from memory and notices that the outcome is negative (e.g., 3−6 = −3), the model will add 10 to the upper term, retrieve a new fact (13−6 = 7), and store in its problem state that a ‘borrowing’ is in progress. The model will then check the problem state every time the subtraction task is resumed. If a ‘borrowing’ is in progress, the model first subtracts 1 from the upper term before the initial retrieval is made.

In the easy version of the text entry task, the model perceives the letter and clicks on the corresponding button. In the hard version, the model has to know the target word and the current position within that word. This information is stored in the problem state resource (e.g. “‘university’, 4th letter”). At each step, the model uses this information to determine the next letter. To simulate the spelling processes, we implemented an additional declarative retrieval that links the current position to the next letter. Although this is a very simplified implementation of the spelling process, it was not necessary to model this aspect of the task in more details since no effects of spelling difficulty are to be expected on the problem state. After the model has determined the next letter, it clicks the appropriate button and updates the problem state to reflect that it is one position further in the word.

The listening task was modeled as a third thread. This thread aurally perceives words, retrieves lexical information related to the auditory input from memory, and builds syntactic trees. The same approach was used by [2] to model the classical reading and dictation study of [26], and by [27] and [28] to account for developmental patterns in children's ability to process pronouns. For each incoming word in the auditory module, four processing steps are taken, and two facts are retrieved from memory. This results in about 320 ms processing time per word, fast enough to keep up with the average speaking rate of 359 ms/word in the presented texts (note that the model is capable of listening to speech faster than 320 ms/word, because the auditory module can already start perceiving a word while other cognitive modules are processing the previous word). The process of answering the multiple-choice questions was not modeled, because modeling the comprehension of a question would have required linguistic processing capabilities at a level of complexity that is beyond the scope of the model. However, the model visually parses the questions when they appear on the screen.

The model explains the interference effect in the following way. In the hard – hard condition a problem state is needed for both the subtraction and the text entry task. This means that the contents of the problem state resource have to be replaced on each step in a trial, increasing response times considerably. Because this is only necessary in the hard – hard condition, the model predicts an over-additive effect of task difficulty on response times. The number of errors will also increase with task difficulty, because older and incorrect problem states are sometimes retrieved. As the model does not use the problem state resource for the listening task, no influence of the listening task on problem state interference is predicted.

**Figure 2:**
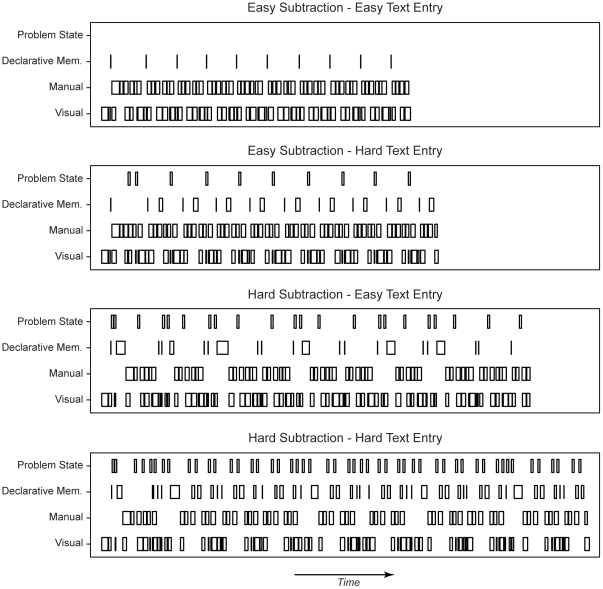
Cognitive resource usage of the model for four trial types. Time goes from left to right; boxes indicate activity of a cognitive resource. Note that only trials are depicted without the listening task.

Figure 2 shows how the model uses cognitive resources over the course of a trial (that is, entering 10 digits and 10 letters). The four panels show four different trial types, ranging from easy subtraction – easy text entry at the top to hard – hard at the bottom (all without the listening task). Boxes indicate that a cognitive resource is in use. A first observation is that the length of the model traces increases with task difficulty: response times increase when the tasks get more difficult. Second, the use of the problem state resource and declarative memory also increases with task difficulty, with an over-additive increase in the hard – hard condition because of the problem state bottleneck. Finally, the use of the manual and visual resources is more or less constant over the different trial types, but gets more spread out in the more difficult conditions. That is, participants have to make the same number of responses in each condition, but because response times are higher these responses are spaced further apart.

The model fit well to the behavioral data from [7]: it fit both the interaction effects in the response times (average R^2^ of .99) and in the accuracy data (average R^2^ of .95; for details see [7]). The same model was used previously to account for the data of two other experiments [7], corroborating the model's explanation of the data. In the next section we will describe how we used the same model to generate fMRI predictions for the current experiment.

#### The fMRI predictions

As mentioned above, the cognitive architecture ACT-R can predict fMRI data, or to be more precise, the BOLD response (e.g., [13], [19]). The modules of ACT-R have been mapped onto specific regions in the brain (see Table 1), and are assumed to predict activation in that region. The most important modules and associated brain regions for the current model are listed in Table 1.

**Table 1:** ACT-R modules and associated brain regions.

ACT-R Module	Brain Region (left hemisphere)	Size (voxels)	Talairach-Tournoux Coordinates	MNI Coordinates
Aural	Sec. auditory cortex (BA 21/22/42)	5×5×5	−45, −22, 9	−48, −21, 7
Manual	Precentral gyrus (BA 3)	5×5×4	−42, −20, 50	−42, −23, 54
Visual	Fusiform gyrus (BA 37)	5×5×4	−41, −61, −9	−43, −60, −16
Problem State	Intraparietal sulcus (BA 7/39/40)	5×5×4	−24, −63, 40	−24, −67, 44
Declarative Memory	Inferior frontal sulcus (BA 45/46)	5×5×4	−42, 23, 24	−43, 24, 25

Voxels are 3×3×3mm. MNI = Montreal Neurological Institute.

ACT-R's modules are not constantly in use during the execution of a model, but operate for short periods of time (in the order of hundreds of ms). The assumption is that when a module is active the BOLD response increases in the associated brain region. The BOLD response of a certain event is modeled by a gamma function, as is customary in fMRI research (e.g., [29]–[31]):
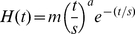
where *t* is the age of the event, *m* determines the magnitude of the BOLD curve, *s* the time scale, and *a* the shape. If *D*(*t*) is a 0–1 demand function that indicates whether a module is active at time *t*, the BOLD activation at time *t* can be calculated by convolving *D*(*t*) with the gamma function:
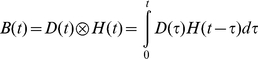
Because the predictions were made before the experiment was run, the gamma function parameters were not fit to data but were set to default ACT-R values (*s* = .75, *a* = 6). The scaling parameter (*m*) was left at 1 (note that therefore only the shape of the predictions is of interest, not the magnitude).

It should be noted that we do not assume that modules in ACT-R cause activation in only these regions, nor that activation in these regions is only due to the associated ACT-R modules. However, these regions have been the best indicators of activation in the ACT-R modules over an extended series of studies (see also http://act-r.psy.cmu.edu/mri and [13]).

The predictions were made using the model described above, adapted for the fMRI-suitable interface of the current experiment. While the experiment is in essence the same as Experiment 3 in [7], some changes were made to the interface to make it suitable for the fMRI scanner. First, in the current experiment, participants were told before each trial what the conditions of the different tasks would be to reduce noise in the fMRI measurements. This was most relevant in the difficult subtraction condition, as in the experiments in [7] participants only discovered during a trial that a subtraction required ‘borrowing’. Second, all responses had to be made using the mouse (instead of the keyboard). Finally the interface was made more compact to reduce eye- and head movements.

**Figure 3:**
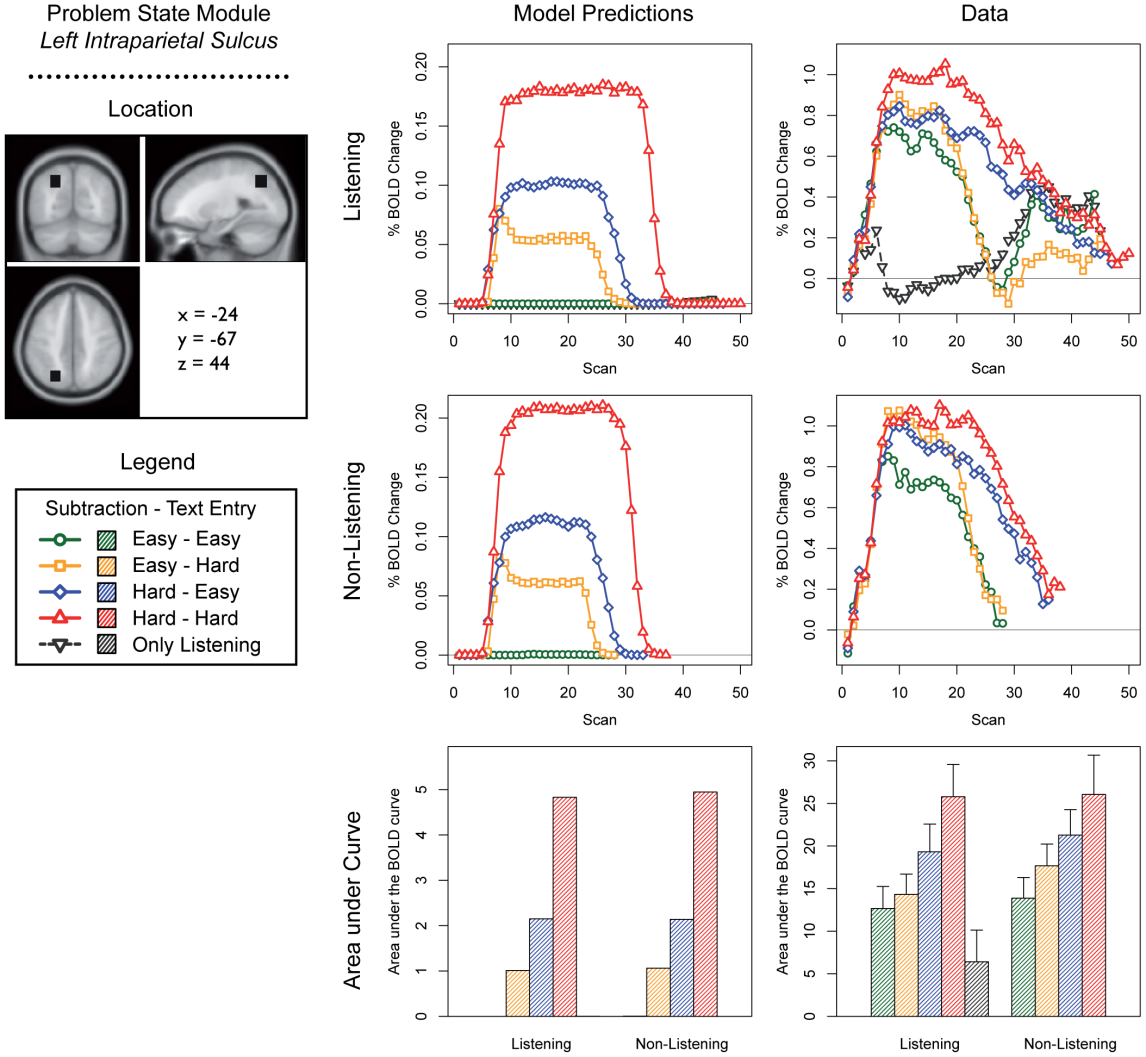
Model predictions and BOLD results for the problem state module. Please note that the green line is hidden behind the black line in the upper left graph.

We discuss predictions for the five most interesting modules of the current model: the problem state module, the declarative memory module, the manual module, the visual module, and the aural module (Figures 3–7; note that these predictions are based on module demand traces similar to those shown in Figure 2). On the left side of each figure the location and the MNI coordinates of the particular module are shown. The three graphs in the center of each figure show the model predictions; the three graphs on the right the fMRI data (which will be discussed in the ‘Results’ section). The four line graphs show the BOLD response over a complete trial (i.e., entering 10 digits and 10 letters, and in the case of the listening task answering the multiple-choice question). The *x*-axis of these graphs represents time in the form of scans (1 scan = 2 seconds); the *y*-axis percent BOLD change (as compared to the average of the first two scans in a trial). The two line graphs at the top show the four conditions when the listening task was present, together with the ‘only listening’ condition. The two line graphs in the middle show the four conditions without the listening task. Finally, the two bar graphs show the area under the curve of the BOLD graphs, indicating the total time a module is active and thus the total activation in a brain area during a trial (as it is sensitive to both the magnitude and the duration of the response, see [6], [32]). We will now discuss the most important predictions; lower-level predictions for each module will be discussed in the results section alongside the experimental results.

**Figure 4 pone-0012966-g004:**
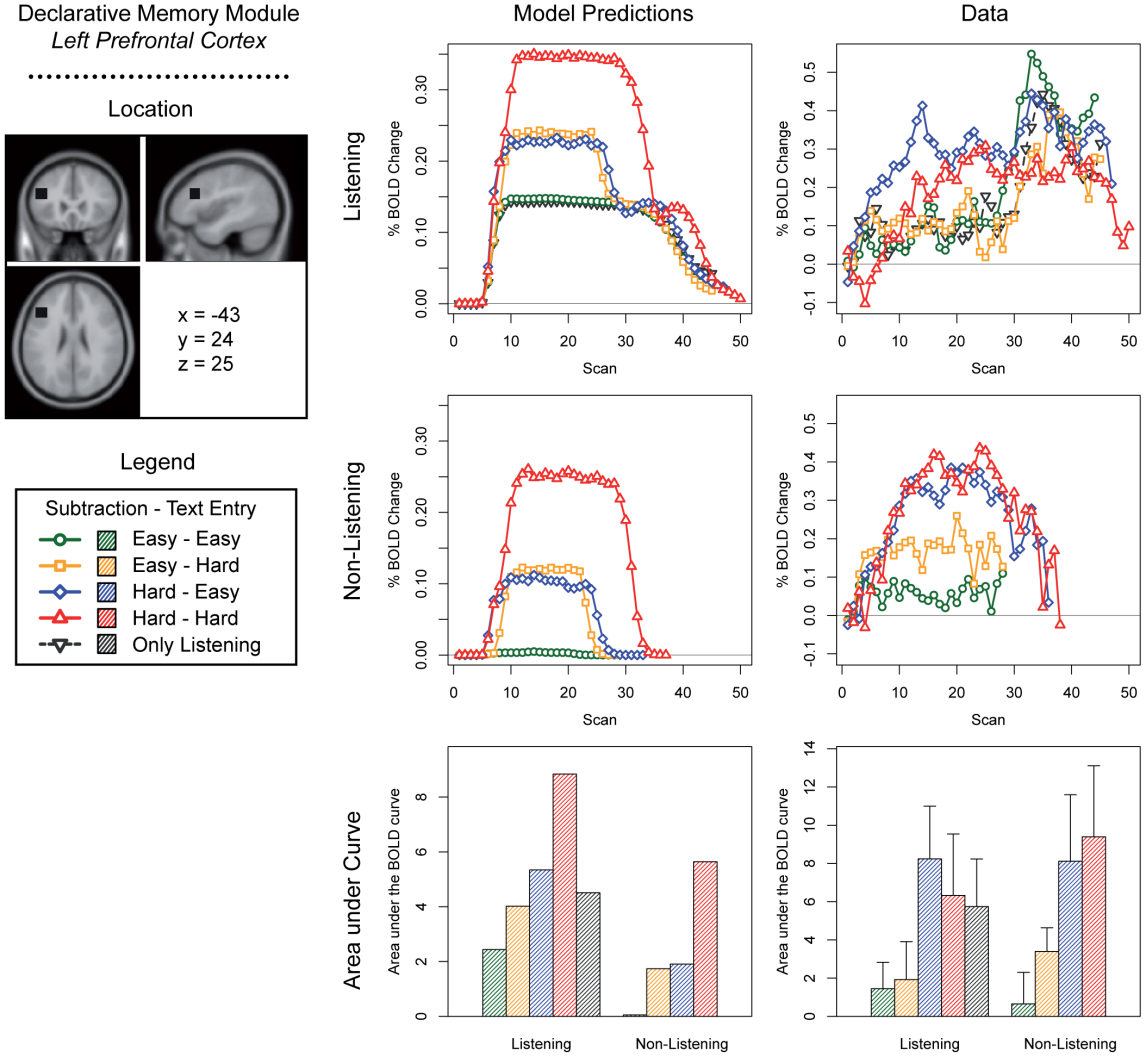
Model predictions and BOLD results for the declarative memory module. Please note the green line hidden behind the black line in the upper left graph.

**Figure 5 pone-0012966-g005:**
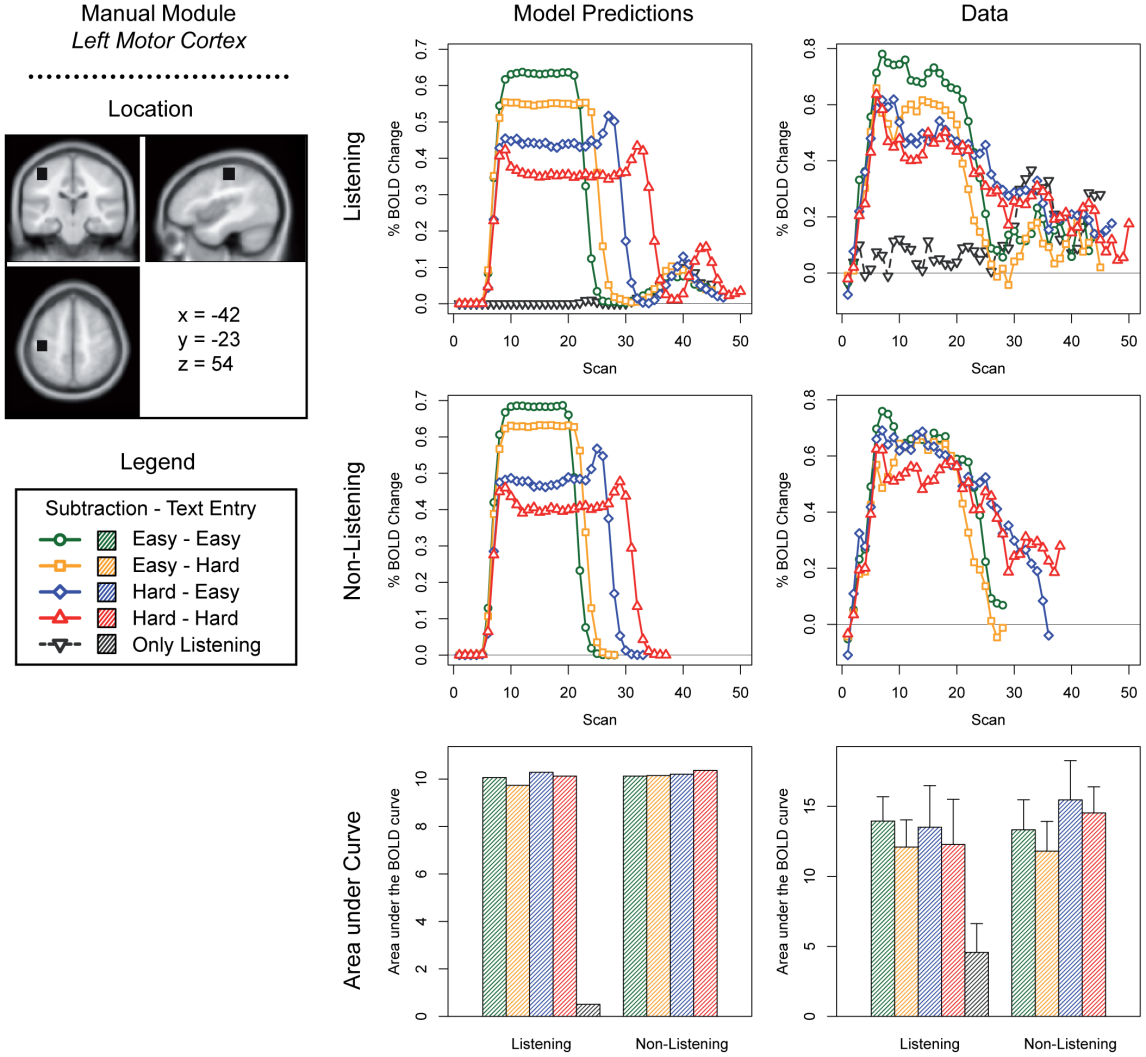
Model predictions and BOLD results for the manual module.

**Figure 6 pone-0012966-g006:**
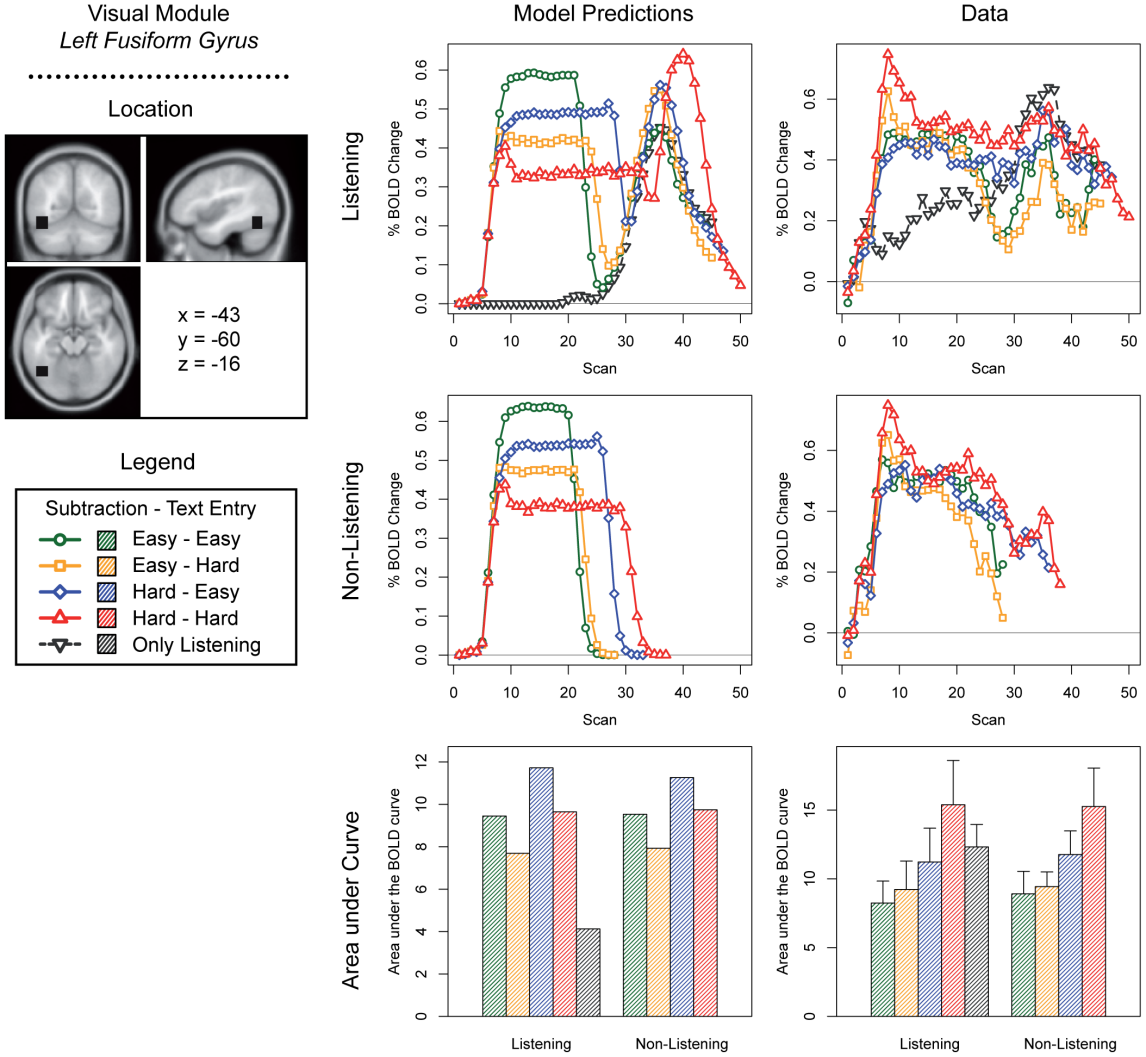
Model predictions and BOLD results for the visual module.

**Figure 7:**
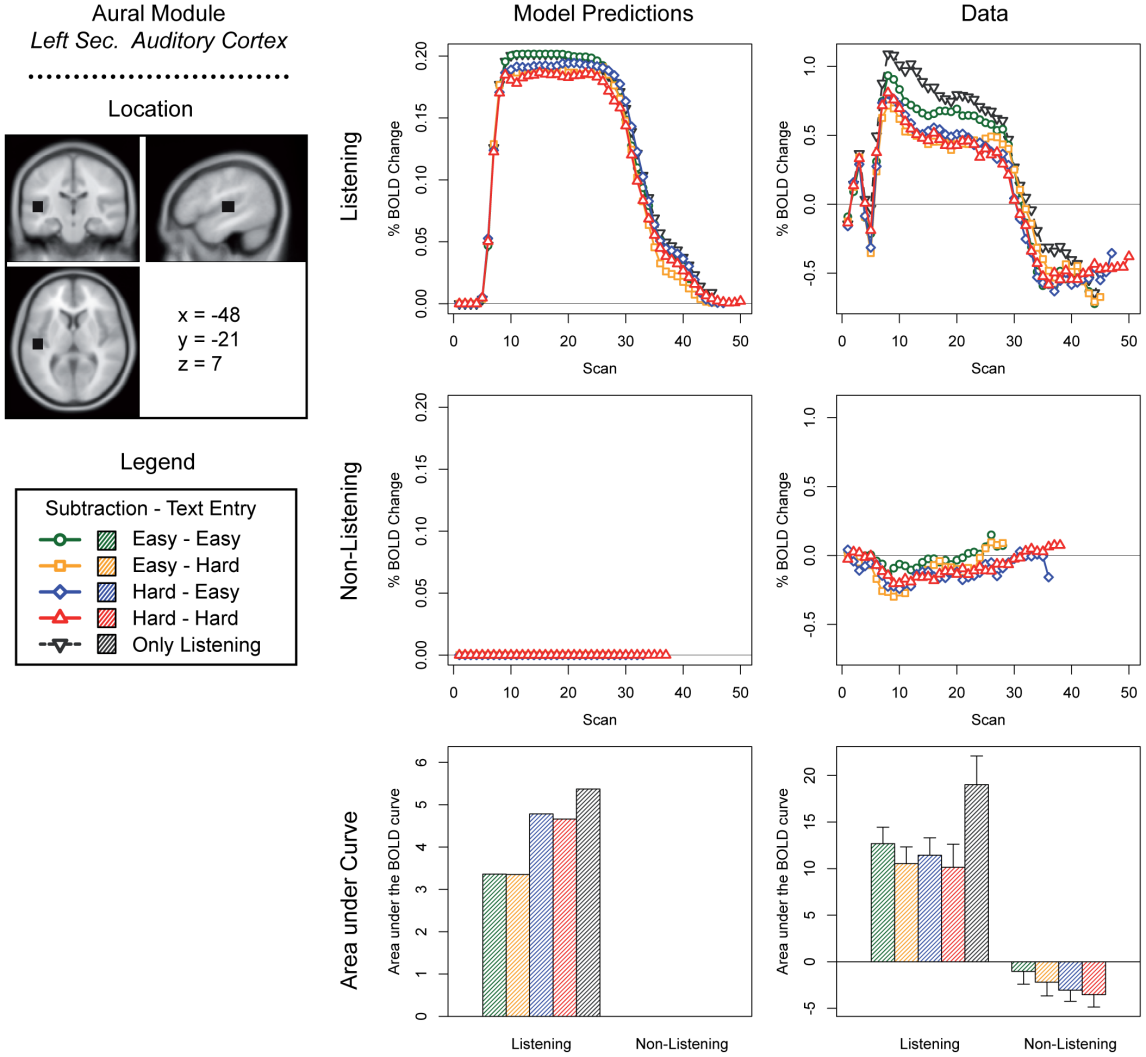
Model predictions and BOLD results for the aural module.

The experiment and the model were developed to investigate the problem state bottleneck. The most important prediction is therefore related to the problem state resource and its associated brain area, the intraparietal sulcus. The model claims that the problem state has to be swapped at every step in a trial in the hard – hard condition. In the other conditions, the problem state is either not used at all (the easy – easy condition), or used only for one of the tasks (easy – hard and hard – easy). Therefore, the model predicts no BOLD activity in the easy – easy condition, intermediate levels in the easy – hard and hard – easy conditions, and the most activity in the hard – hard condition (Figure 3; cf. Figure 2). In fact, as the area under the curve reflects the total time that a module is active, an over-additive interaction effect is predicted in the intraparietal sulcus. Because the declarative memory module is used to retrieve the old problem state on each step in a trial in the hard – hard condition, a similar interaction effect is predicted for the declarative memory module (Figure 4).

For the manual module (Figure 5), opposite patterns are predicted, with highest BOLD peaks occurring in the easier conditions. This may seem odd, because participants have to make the same number of responses in each condition. However, because response times are longer in the more difficult conditions, the BOLD response has more time to decay between each response (see also Figure 2). Therefore, the curves are lower but broader in the more difficult conditions, and higher and narrower in the easier conditions: the area under the curve is equal in all conditions. A similar pattern is predicted for the visual module (Figure 6). However, more visual activity is predicted for the hard subtraction condition than for the easy subtraction condition, because the model has to look multiple times at the digits to process the ‘borrowings’. With respect to the aural module (Figure 7), the model obviously predicts no activity in the non-listening conditions, and sustained levels of activity in the listening conditions.

To summarize, the model does not predict a general increase in BOLD response with task difficulty; instead, it predicts *lower but more persistent* activation levels for the more difficult conditions in the visual and manual modules, and *higher and more persistent* activation levels for the more difficult conditions in the problem state and declarative memory modules. In the next section the fMRI experiment is described that was carried out to test those predictions.

## Methods

### Experimental Procedures

The design of the experiment is described in ‘A Priori Predictions – The Triple Task’ and Footnote 3. The participants performed the experiment in three sessions. The first session was a practice session, in which the participants were familiarized with the task, and trained for about 30 minutes. The next day the first of two fMRI sessions of about 90 minutes took place, followed by the second fMRI session a few days later (on average 3.3 days after the first session, range 1–9 days). The two fMRI sessions were identical.

#### Participants

Thirteen students of Carnegie Mellon University participated in the experiment. Three of them had to be excluded: one for falling asleep in the MRI scanner, one for ignoring the listening task, and one for fMRI recording problems, which leaves 10 complete datasets (3 women, average age 21.9, range 19–28, right-handed). All participants had normal or corrected-to-normal vision and normal hearing. Written informed consent as approved by the Institutional Review Boards at Carnegie Mellon University and the University of Pittsburgh was obtained before the experiment. Participants received US$ 100 compensation for performing the practice session and the two experimental sessions.

#### Stimuli

The stimuli for the subtraction task were generated anew for each participant. The subtraction problems in the hard version always featured six ‘borrowings’, and resulted in 10-digit answers. The 10 letter words for the hard version of the text entry task were handpicked from a list of high-frequency English words (CELEX database) to ensure that similarities between words were kept at a minimum. These stimuli were also used in the easy text entry task, except that the letters within the words were scrambled (under the constraint that a letter never appeared twice in a row). Thus, participants were presented pseudo-random sequences of letters that they had to enter one-by-one in the easy condition. By scrambling the words, we controlled for letter-based effects, while preventing the use of strategies to predict the next letter.

The audio recordings and questions for the listening task were taken from four English listening comprehension exams (university entrance-level in the Netherlands, VWO Engels 2004–2007, Cito Arnhem). The story length ranged between 26 and 72 seconds (*M* = 52.6, *SD* = 9.7). The multiple-choice questions, which participants only saw after hearing the text, had three options. These questions could be answered without making inferences, but did require attention for the complete duration of the story.

During the practice session, the experiment was presented full screen on a 17″ monitor. The width of the interface measured 20 cm; the overall height 9 cm (see also Figure 1). Participants were sitting at a normal viewing distance, about 75 cm from the screen. The stories were presented via speakers, of which participants could control the volume using the keyboard. During the experimental sessions, the experiment was projected on a screen in the MRI scanner, allowing the participants to view the experiment via a set of mirrors attached to the head coil. The interface was operated through a normal computer mouse using the right hand. The listening task was presented via fMRI-compatible headphones, reducing scanner noise to allow the participants to hear the stories. Participants could change the volume of the stories using the mouse wheel.

#### Procedure

Each trial started with the presentation of a fixation cross, followed by two colored circles indicating the difficulty levels of the tasks (on the left for the subtraction task, on the right for the text entry task; a green circle for easy, a red circle for hard, two open circles for the ‘only listening’ condition). If the listening task was present, a short beep sounded when the circles were displayed. The circles stayed on the screen for 5 seconds, followed by a fixation cross for 1 second. Afterwards, the subtraction and text entry tasks appeared and, in case of the listening task, the story started. Participants always begun with the subtraction task, and then alternated between the two tasks. After completing both tasks, a feedback screen was shown for 3 seconds, indicating how many letters / digits were entered correctly. After the feedback screen and after the story was finished, the multiple-choice question was displayed. When the participants clicked on an answer, a feedback screen was shown for 4 seconds. The experiment was slow event-related, with trials separated by long breaks whose duration was sampled from a uniform distribution between 13 and 17 seconds. The onset of the circles as well as the onset of the tasks was synchronized with the beginning of a volume acquisition.

The practice session consisted of 13 single task trials, followed by a block of 9 multitask trials: all combinations of subtraction and text entry in combination with the listening task (4 trials: easy-easy, hard-easy, easy-hard, and hard-hard), without the listening task (4 trials), and one ‘only listening’ trial. Both experimental sessions consisted of 5 multitask trial blocks and of one practice block at the start of a session, to re-familiarize participants with the task (this was performed during the acquisition of structural images, allowing the participants to get habituated to the environment and to adapt the listening-volume before the experimental trials). Trials were randomized within a block; stimuli were randomized over the two experimental sessions. The complete experiment (two sessions) consisted of 90 experimental trials. After each block participants could take a short break.

### fMRI Procedures and Preprocessing

The fMRI data were collected with a Siemens 3T Allegra Scanner using a standard radio frequency head coil. Each functional volume existed of 34 axial slices (3.2 mm thickness, 64×64 matrix, 3.125×3.125 mm per voxel), acquired using echo-planar imaging (2000 ms TR, 30 ms TE, 79° flip angle, 200 mm field of view, 0 slice gap, with AC-PC on the 11^th^ slice from the bottom). Functional acquisition was event-related; scanning onset was synchronized with stimulus onset as described above. Anatomical images were acquired using a T1-weighted spin-echo pulse sequence at the same location as the functional images but with a finer resolution (3.2 mm thickness, 200 mm field of view, 256×256 matrix, 0.78125×0.78125 mm in-plane resolution).

The data were analyzed using SPM5 (Wellcome Trust Centre for Neuroimaging, http://www.fil.ion.ucl.ac.uk/spm/). This included realigning the functional images, coregistering them with the structural images, normalizing the images to the MNI (Montreal Neurological Institute) ICBM 152 template, and smoothing them with an 8 mm FWHM Gaussian kernel. The MarsBaR toolbox [33] was used to extract the time course information in predefined regions.

## Results

We will first discuss the behavioral results, followed by the fMRI region-of-interest results. An exploratory fMRI analysis was also performed, which confirmed the existence of peaks of activations in the standard ACT-R regions-of-interest (see Text S1 and Tables S1, S2, and S3 for more details). All reported *F*- and *p*-values are from repeated measure analyses of variance (ANOVAs), all error bars depict standard errors, effects were judged significant when a .05 significance level was reached, and accuracy data were transformed using an arcsine transformation before performing ANOVAs.

### Behavioral Results

Outliers in response times were eliminated by means of a two step procedure. First, response times faster than 250 ms and slower than 10,000 ms were removed. Then, data exceeding 3 standard deviations from the mean per condition per participant were excluded. Overall, 2.4% of the data was discarded. Table 2 (text entry) and Table 3 (subtraction) summarize the results.

**Table 2:** ANOVA results of the text entry task.

	Response Times			Accuracy		
*Source*	*F(1,9)*	*p*	*η_p_^2^*	*F(1,9)*	*p*	*η_p_^2^*
Listening	10.69	.010	.54	10.37	.010	.54
Subtraction	32.43	<.001	.78	8.72	.016	.49
Text Entry	5.67	.041	.39	32.17	<.001	.78
Listening×Subtraction	<1	-	-	1.60	.24	.15
Listening×Text Entry	<1	-	-	<1	-	-
Subtraction×Text Entry	12.08	.007	.57	10.35	.01	.53
Listening×Sub.×Text Entry	<1	-	-	<1	-	-

Subtraction = Subtraction Difficulty, Text Entry = Text Entry Difficulty.

**Table 3:** ANOVA results of the subtraction task.

	Response Times			Accuracy		
*Source*	*F(1,9)*	*p*	*η_p_^2^*	*F(1,9)*	*p*	*η_p_^2^*
Listening	1.58	.24	.15	<1	-	-
Subtraction	83.82	<.001	.90	80.96	<.001	.90
Text Entry	5.40	.045	.38	4.34	.067	.33
Listening×Subtraction	<1	-	-	1.44	.26	.14
Listening×Text Entry	<1	-	-	2.81	.13	.24
Subtraction×Text Entry	2.70	.13	.23	14.05	.005	.61
Listening×Sub.×Text Entry	1.47	.26	.14	<1	-	-

Subtraction = Subtraction Difficulty, Text Entry = Text Entry Difficulty.

#### Results

**Figure 8:**
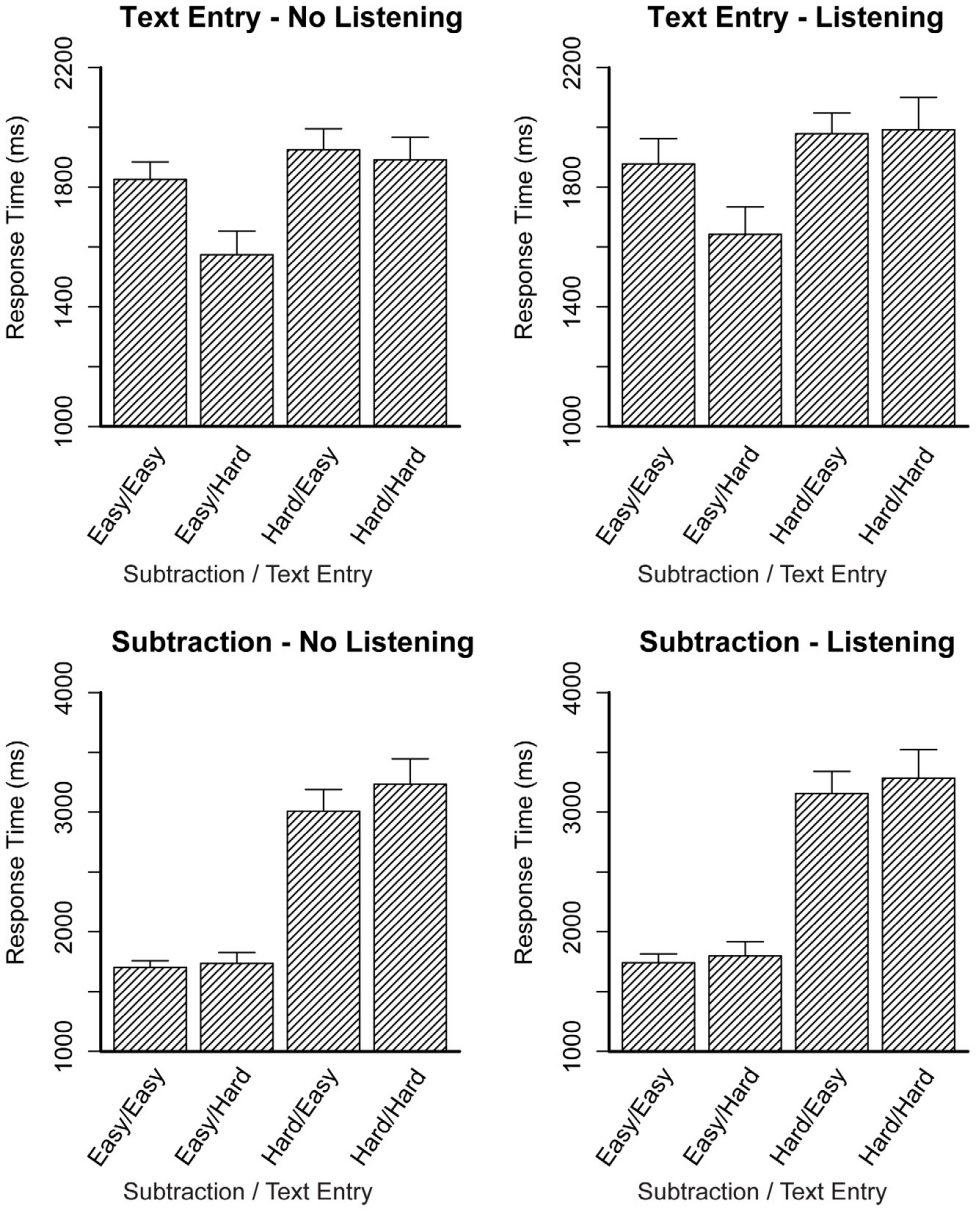
Response times on the subtraction and text entry tasks. Error bars represent standard errors.

Figure 8, upper panels, shows the response times on the text entry task, on the left without and on the right in combination with the listening task. A response time on the text entry task was defined as the time between entering a digit in the subtraction task and entering a letter in the text entry task. The first responses of each trial were removed (per task), as they might contain ‘start-up’ effects. An ANOVA showed that all three main effects were significant (see Table 2), indicating that response times decreased with Text Entry Difficulty, but increased with Subtraction Difficulty and Listening. The interaction between Subtraction Difficulty and Text Entry Difficulty also reached significance, which is due to the increased response times in the hard-hard condition, as was predicted. The three-way interaction did not reach significance.

The lower panels of Figure 8 show the response times on the subtraction task. This is the time between clicking a button in the text entry task and entering a digit in the subtraction task. Again, the first response of each trial was removed. Only the main effects of Subtraction Difficulty and Text Entry Difficulty reached significance (see Table 3), showing an increase in response times for both effects. The interaction of Subtraction Difficulty and Text Entry Difficulty did not reach significance, nor did any effects involving the listening task.

**Figure 9:**
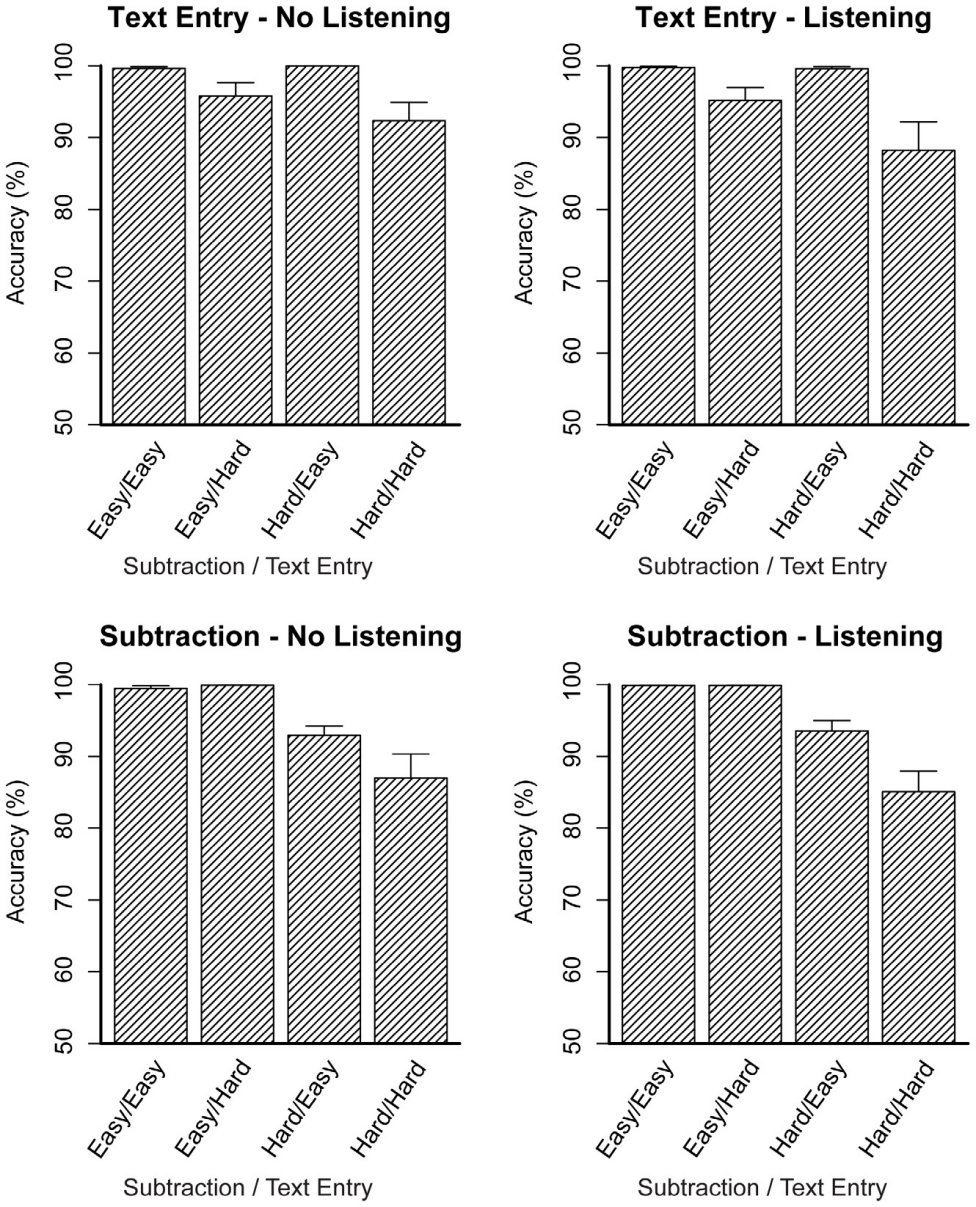
Accuracy on the subtraction and text entry tasks. Error bars represent standard errors.

Figure 9 shows the accuracy on the subtraction and text entry tasks (the ANOVA results are listed in Table 2 and 3). The two top panels show the accuracy on the text entry task. All three main effects reached significance, all three indicating a decrease in accuracy. As predicted, when both the subtraction and the text entry task were hard, accuracy decreased even more, which is shown by the significant interaction between Subtraction Difficulty and Text Entry Difficulty. The other effects did not reach significance. The lower panels of Figure 9 show the accuracy on the subtraction task. The main effect of Subtraction Difficulty was significant, as was the interaction between Subtraction Difficulty and Text Entry Difficulty. The other tests did not reach significance.

**Figure 10:**
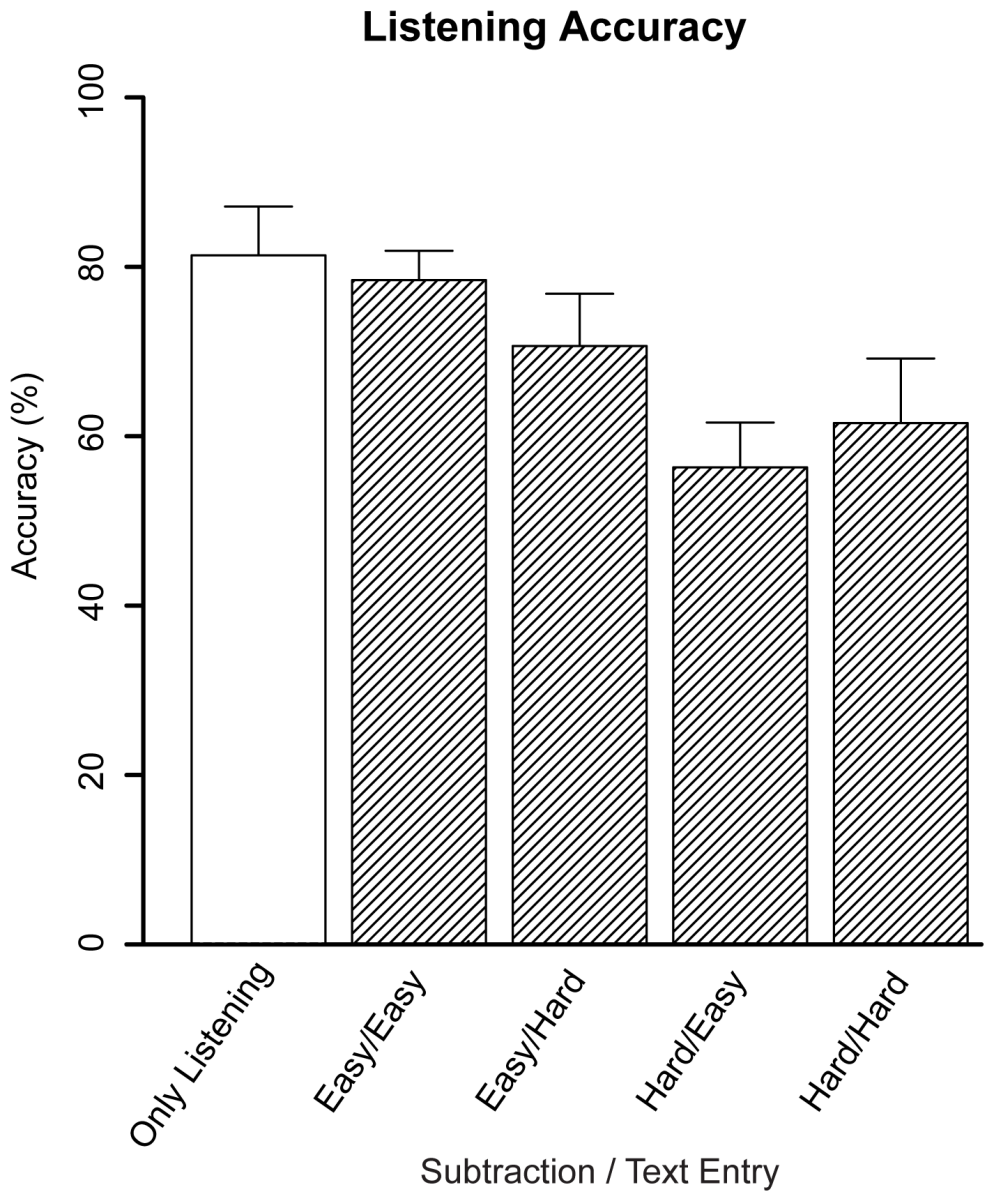
Accuracy on the listening task. Error bars represent standard errors.

Figure 10 shows the accuracy on the listening task. One of the stories was removed because participants' accuracy was at chance level. Only the main effect of Subtraction Difficulty reached significance (*F*(1,9) = 18.09, *p* = .002, *η_p_^2^* = .67), caused by a decrease in accuracy when subtraction was hard. The other effects were not significant (Text Entry Difficulty: *F*<1; Subtraction Difficulty×Text Entry Difficulty: *F*(1,9) = 1.29, *p* = .28, *η_p_^2^* = .13).

#### Discussion

The results were as expected: the interaction effect of Subtraction Difficulty and Text Entry Difficulty was significant for the response times of the text entry task and for the accuracy scores of both tasks. Thus, when a problem state was required for both tasks (the hard - hard condition), response times increased and accuracy decreased, as was predicted by the model. The fact that this interaction did not reach significance (*F*(1,9) = 2.7, *p* = .13, *η_p_^2^* = .23) for the response times of the subtraction task is probably due to the lower number of participants than in previous experiments, in which the effect was always significant [7]. Furthermore, compared to previous experiments, response times were slightly higher. This difference is probably due to performing the experiment in the scanner and using the mouse.

The pattern of response times of the text entry task was slightly different than in the previous experiment (Experiment 3 of [7]): response times were lower in the hard version of the text entry task than in the easy version. The explanation is that participants have to do two different actions to determine the next letter to type in the text entry task: in the easy version they have to look at the letter that they need to type, and in the hard version they have to mentally determine the next letter to type given the word and position. In earlier experiments these two actions happened to take approximately the same amount of time, but in the current experiment the action for the easy version of the task turned out to be slower, probably due to the slightly different interface that we used in this experiment. We have observed similar effects before, for instance in Experiment 2 of [7].

To ensure that the fMRI experiment is comparable to our earlier studies, we ran the same experiment outside the scanner. This yielded similar results as reported in [7], including the decrease of response times for the hard text entry task, indicating that the observed differences are not due to the minor changes in the task interface. That is, the fact that the interaction effect did not reach significance is probably caused by the low number of participants, and the slightly different pattern of results by performing the task lying in the scanner and operating the mouse in this setup. This suggests that the experiment still taps the same underlying cognitive constructs and that it is therefore comparable to our previous studies. For details see Text S2, Figures S1 and S2, and Tables S4 and S5.

The model did not predict the effect of the listening task on the text entry task, neither the small increase in response time nor the small decrease in accuracy. As this is not the focus of the current paper, we did not pursue this issue further. The effect of the subtraction task on the listening task accuracy, Figure 10, is explained by the model: When the subtraction task is hard, there is a high demand for declarative memory, causing the model to not process all words of the listening task (for which declarative memory is also required). This could then lead to more mistakes in answering the questions (see [7] for a more extensive discussion of this issue).

### fMRI Results: Regions-Of-Interest

To analyze the effects in the predefined regions, we first transformed the Talairach-Tournoux coordinates used in previous ACT-R/fMRI papers (e.g., [13]) to the MNI coordinates reported in Table 1 using a non-linear mapping (see [34]). The smoothed functional images were proportionally and grand mean scaled (with a grand mean of 100) using SPM. The BOLD response was then calculated as percent signal change as compared to the first two scans of a trial. Trials belonging to the same participant, brain area, and condition were averaged together. Because the area under the curve reflects the total activity of a brain area (see [6], [32]), we entered this value into an ANOVA. We only took the area between the start of a trial and the behavioral feedback screens into account, because the tails of the BOLD curves contain the multiple-choice questions. These could obscure the results and were not included in the ACT-R model (except for the passive act of reading the words on the screen). Table 4 contains the results; Table S6 shows which scans were taken into account for the different conditions.

**Table 4:** ANOVA results of the area under the curve of the regions-of-interest.

Problem State Module – Intraparietal Sulcus						
	*With Listening*			*Without Listening*		
*Source*	*F(1,9)*	*p*	*η_p_^2^*	*F(1,9)*	*p*	*η_p_^2^*
Subtraction	60.20	<.001	.87	20.89	.001	.70
Text Entry	6.49	.031	.42	10.39	.010	.54
Subtraction×Text Entry	5.15	.049	.36	<1	-	-

Declarative Memory Module – Prefrontal Cortex						
	*With Listening*			*Without Listening*		
*Source*	*F(1,9)*	*p*	*η_p_^2^*	*F(1,9)*	*p*	*η_p_^2^*
Subtraction	11.73	.008	.57	7.53	.023	.46
Text Entry	<1	-	-	9.81	.012	.52
Subtraction×Text Entry	2.03	.19	.18	<1	-	-

Manual Module – Motor Cortex						
	*With Listening*			*Without Listening*		
*Source*	*F(1,9)*	*p*	*η_p_^2^*	*F(1,9)*	*p*	*η_p_^2^*
Subtraction	<1	-	-	3.51	.09	.28
Text Entry	1.85	.207	.17	<1	-	-
Subtraction×Text Entry	<1	-	-	<1	-	-

Visual Module – Fusiform Gyrus						
	*With Listening*			*Without Listening*		
*Source*	*F(1,9)*	*p*	*η_p_^2^*	*F(1,9)*	*p*	*η_p_^2^*
Subtraction	11.52	.008	.56	11.12	.009	.55
Text Entry	3.00	.117	.25	4.06	.075	.31
Subtraction×Text Entry	2.42	.154	.21	1.35	.276	.13

Aural Module – Secondary Auditory Cortex						
	*With Listening*			*Without Listening*		
*Source*	*F(1,9)*	*p*	*η_p_^2^*	*F(1,9)*	*p*	*η_p_^2^*
Subtraction	<1	-	-	3.32	.102	.27
Text Entry	2.00	.191	.18	1.39	.269	.13
Subtraction×Text Entry	<1	-	-	<1	-	-

#### Results

The most important prediction of the model was an over-additive interaction effect in the intraparietal sulcus, reflecting the problem state bottleneck. Figure 3 shows the results in the intraparietal sulcus: most activation is indeed observed for the hard – hard condition. The ANOVA of the area under the curve shows that the interaction between Subtraction Difficulty and Text Entry Difficulty is significant in combination with the listening task, but not without it (see Table 4). Furthermore, the main effects of Subtraction Difficulty and Text Entry Difficulty are significant with and without the Listening task. The model prediction that there is no activation for the easy – easy condition did not come true, but the prediction that the problem state resource is not used for the listening task – except for answering the multiple-choice question – is reflected by the data.

Figure 4 shows the results of the prefrontal cortex, associated with the retrieval module. For this region the model also predicted an over-additive interaction effect of Subtraction Difficulty and Text Entry Difficulty, which was not found in the data. The model also predicted main effects of both Subtraction Difficulty and Text Entry Difficulty and these effects were indeed found (Text Entry Difficulty was only significant without the listening task, Subtraction Difficulty both with and without listening).

In contrast to the problem state and declarative memory modules, we expected a higher BOLD response peak for the easier conditions in the manual and visual areas. Indeed, in the motor cortex – associated with the manual module – the BOLD curve reached its highest activation levels in the easy – easy condition (Figure 5). The more difficult the condition, the lower and broader the activation curves. The model predicted no effects on the total activity; this was confirmed by the data.

At first sight, the match between model and empirical data for the fusiform gyrus (Figure 6), associated with the visual module, seems less convincing. However, a more careful analysis shows that the same patterns are observed in both model and data. The model predicted an effect of Subtraction Difficulty on activation in the fusiform gyrus, as the digits have to be visually attended to multiple times in the hard condition to solve the ‘borrowings’. This is confirmed by the ANOVA that compared the area under the curve between the easy and difficult conditions. While the model also predicted a small decrease of visual activation in the hard text entry conditions (because in the hard condition the word only had to be read at the first step of a trial, while in the easy condition a letter had to be processed at each step of a trial), this was not found in the data. Finally, the model predicted a peak of activation around scan 40 caused by reading the multiple-choice questions; this was reflected in the data.

Figure 7 illustrates the results for the auditory cortex. As expected, when the listening task was not present, the BOLD response was absent. When the listening task was present, on the other hand, a clear BOLD response was found. The model predicted this effect, and additionally predicted a small effect of condition. The cause of this effect is that to process each word that the model hears, it has to retrieve multiple facts from declarative memory. In the more difficult subtraction and text entry conditions, these tasks also make heavy demands on declarative memory. When declarative memory is busy, the model can sometimes not process a word right away, which results in missing some words in the auditory stream. Thus, the more difficult the subtraction and text entry conditions, the higher the demands on declarative memory, the more words are missed, and the lower the predicted BOLD response for the secondary auditory cortex (as this region reflects processing auditory information, not passive listening). A similar effect seems to be present in the data, but did not reach significance.

#### Discussion

The atypical prediction that the more difficult conditions would show *lower* but broader activation curves in the visual and manual regions, and *higher* and broader curves in the problem state and declarative memory conditions, was confirmed by the data. Furthermore, the over-additive interaction effect in the problem state region was present in the fMRI data (in combination with the listening task), supporting the theory that the problem state bottleneck is localized in the intraparietal sulcus. This interaction effect was not found in the declarative memory region (see the General Discussion for an extensive discussion of this issue). In the aural region the predictions were confirmed in general: a BOLD response in the listening conditions, mediated by the conditions of the subtraction and text entry tasks. However, these effects did not reach significance.

## Discussion

The current study was performed to investigate the neural correlates of problem states and the problem state bottleneck, and to validate our theory using neuroimaging data. First, we generated *a priori* fMRI predictions for five brain areas using our model, which were subsequently tested in an experiment. This resulted in two main predictions: (1) an over-additive interaction effect in the problem state region (the intraparietal sulcus) and in the declarative memory region (a part of the prefrontal cortex), and (2) lower and broader BOLD curves for the more difficult conditions in the manual and visual regions, and higher and broader BOLD curves for the more difficult conditions in the problem state and declarative memory regions. The first prediction came true for the problem state region, but not for the declarative memory region, while the counter-intuitive second prediction was confirmed by the experiment.

In general, the model's fMRI predictions for this complex task were accurate. The paper focuses mainly on the overall BOLD response in the regions (area under the curve). The figures also report the time course of the BOLD response over a trial together with the corresponding model predictions. Here the fit between the scan-by-scan data points and the model is more modest, which can be explained by the fact that we made *a priori* predictions, and did not try to fit the curves post-hoc. A number of factors might be called into question. First, ACT-R uses only a simple gamma function, identical for every module, to predict the BOLD response in each region. However, the biological hemodynamic response function is more complex than that, and varies in different parts of the brain (e.g., [35]). Choosing different functions and fitting their parameters for each region separately would probably result in a better model-data match. Second, due to the duration of our experimental paradigm, only a relatively small number of observations for each condition was available for each participant. This small number of observations might not be able to cancel the scan-to-scan variations of noise in the MRI signal, thus making the true shape of the observed BOLD curves difficult to estimate. Third, the model might be underestimating some trial-by-trial variability in the subjects' responses. In particularly long trials, the BOLD response in a region might cumulate over the interval between trials and carry over to the scans chosen as a baseline for the successive trial. The fact that certain BOLD curves (especially in Figures 4 and 5) do not return to baseline suggests that this kind of contamination was indeed occurring, possibly corrupting the true shape of the BOLD curves.

It should be noted that, while all these factors can affect the shape of the BOLD response, none of them should significantly impact our predictions on the relative magnitudes of the areas under the curve. Therefore we choose to focus on the predictive power of the model and its principal predictions. In combination with the behavioral evidence that we gathered before [7], the observed global effects on the BOLD response suggest that the hypothesized existence of a problem state bottleneck can explain the interference effects in the data.

The most important prediction of the model was an over-additive interaction effect in the problem state region. While this effect was indeed present in the data in combination with the listening task, it did not reach significance in the trials without the listening task. The main reason for this is that while the model predicted no activity in the problem state region for the easy-easy condition, the experimental data does show increased activity in this condition. One possible explanation is that the observed activity was caused by visually processing the stimuli, as the same parietal region is known to be involved in visual-spatial processing (e.g., [36]). Not only would this lead to an effect in the easy-easy condition, but also obscure the effects in the other conditions. In combination with non-linear properties of the BOLD response (e.g., [37]–[39]), this could explain why we did not observe the interaction effect here, especially taken into account the relatively low number of participants. Another possibility is that participants use their problem state resources in the easy-easy condition to represent information, even if this is not required by the task. This would lead to neural activity in the easy-easy condition, again canceling the interaction effect. The additional load of the listening task could have prevented the use of problem state resource (see, for similar effects, [25]), which could explain why we did find the interaction effect in the context of the listening task. However, as the model has successfully accounted for data of three experiments [7], we do believe that the basic mechanisms of the model are sound, and decided against post-hoc changes to the model.

The prefrontal region corresponding to the declarative memory module exhibits the predicted main effects of subtraction difficulty and text entry difficulty (except for Text Entry Difficulty when the listening task was present). This supports the hypothesis that this predefined region indeed represents an area involved in the processing of declarative memory elements (such as subtraction facts). However, we did not find the predicted interaction effect. The interaction effect was supposed to be caused by encoding of problem states (on top of retrieving subtraction facts). Even though the predefined area is known to be active when intentionally encoding facts and even when unintentionally encoding facts (e.g., [40]), the experiment did not provide evidence that it is actually used to encode suspended problem states. Therefore, either this region's contribution to the processing problem states was too weak to impact the BOLD signal, or the retrieval of suspended problem states is controlled by a different region.

With respect to the first option (i.e., the contribution to the signal being too weak) one must note that the predictions made by our model were based on the assumption that both retrieving a previous problem state from declarative memory and swapping it into the problem state module require some measurable cost in terms of time. When the model was fit to the behavioral data of [7], these two costs had to be estimated together, with no possibility of disentangling them. However, it is conceivable that the retrieval time for a problem state is very short, and that most of the time is due to the swapping process. Under such circumstances, the model would still predict the over-additive effects of task difficulty for the problem state region, but not for the retrieval region. In fact, there are at least two reasons why the retrieval time for problem states should be very short. The first reason is recency: the problem state that needs to be retrieved has been swapped out of its module only a few seconds before, and it is probably still active in memory. Second, the retrieval of appropriate problem states can be easily cued by task-relevant, on-screen information. In both cases, there is no reason to expect a significant effect of problem state retrievals on the prefrontal region. In fact, the pattern of data in Figure 5 (lower half) suggests that main factor affecting the response of the retrieval region is the difficulty of the subtraction task. Thus, although the interaction effect in the PFC was an *a priori* prediction of our model, it was not an inevitable prediction and its lack does not undermine the plausibility of our framework.

On the basis of previous ACT-R/fMRI research, the model predicted that the problem state resource – and thus the effect of the bottleneck – is located in the intraparietal sulcus. This notion is supported by the current results: the predicted interaction effect caused by the problem state bottleneck was found in this region. This region is part of the fronto-parietal network that is consistently found in neuro-imaging studies of working memory. While the intraparietal sulcus is mostly implicated in spatial working memory and spatial attention tasks, it is also known to be responsible for object and verbal working memory (among other regions, e.g., [41], [42]). In our study, the problem states did not contain spatial information, and therefore confirms a more general role of the intraparietal sulcus for working memory.

In the hard subtraction task the problem state resource contained numerical information, that is, information whether a ‘borrowing’ is in process. It is not surprising that this leads to increased activation in the intraparietal sulcus, as the horizontal part of the intraparietal sulcus is one of the three circuits for numerical processing as identified by [43]. In the hard text entry task, the problem state is used to maintain verbal information. Brodmann Area 40, a region bordering on the intraparietal sulcus, is known to be involved in verbal working memory, specifically in maintaining verbal working memory (e.g., [44]), and it is thus also not surprising that this region is involved in maintaining the problem state for the text entry task. While slightly different regions are implicated for storage of different kinds of information, this study suggests that maintaining more than one problem state of any kind at a time results in significant interference.

The current results also seem to suggest that the problem state is modality-specific. The subtraction and text entry task elicit activation in the intraparietal sulcus even when they are easy (while a problem state is not required), and interfere with each other in the hard – hard condition. The listening task, on the other hand, hardly causes activation in the intraparietal sulcus (as shown by the ‘only listening’ condition), nor does it cause multitasking interference. As the listening task is the only non-visual task, this could imply that the intraparietal sulcus is only involved in maintaining visual problem states (cf. [45]).

In the current mapping scheme of ACT-R processes to brain regions, the problem state predicts activation as a function of problem state transformations, but not in reaction to storing problem states. This may seem odd, as storing problem states should also have metabolic costs. In practice, however, the two processes of storing and manipulating are difficult to separate, (as storing always follows a problem state manipulation) and previous research (see e.g., [13]) has led to estimated costs for transformations only, assuming that representations persist at no additional metabolic cost. Therefore, we decided to keep our model as parsimonious as possible and not to introduce ad-hoc estimates of the storing costs for problem states.

Our model is based on threaded cognition [2], [5], [23], a theory of multitasking that assumes multiple central and peripheral processing bottlenecks. This in contrast to for instance the EPIC theory [46], which assumes only peripheral bottlenecks, and the central bottleneck theory of [47], which assumes only a single central bottleneck. While brain evidence for a central bottleneck in the frontal lobes has been reported before (e.g., [48]), the current fMRI results give evidence for an additional central bottleneck located in the intraparietal sulcus, corroborating multiple-bottleneck theories.

In conclusion, this study lends additional support to the notion of the problem state bottleneck. This bottleneck can cause considerable interference not only in concurrent multitasking – as most bottlenecks – but also in sequential multitasking: When multiple alternating tasks need to store intermediate results, the problem state bottleneck will cause significant interference. Take for instance the prototypical example of taking a phone call while working on a paper: if you had a sentence in mind before taking the call, you will almost certainly have forgotten about it after the call.

## Supporting Information

Text S1 Exploratory fMRI Analysis. This text discusses the exploratory fMRI analysis that was performed besides the Region-Of-Interest analyses that are reported in the main text.
(0.04 MB DOC)

Text S2 Behavior results outside the scanner. This text discusses the results of an experiment that we ran outside the fMRI scanner. This experiment was performed to test whether differences between the behavioral results of the current fMRI experiment and a previous experiment are due to performing the experiment in the scanner and the low number of participants, or to the slightly different interface.
(0.04 MB DOC)

Table S1 Exploratory analysis results. Areas with greater activation for Subtraction, Text Entry, and Listening than Subtraction and Text Entry alone (p<.05, FDR corrected, >40 contiguous voxels).
(0.03 MB DOC)

Table S2 Exploratory analysis results. Areas with greater activation for Hard Text Entry than Easy Text Entry (p<.05, FDR corrected, >40 contiguous voxels). SMA = Supplementary Motor Area.
(0.03 MB DOC)

Table S3 Exploratory analysis results. Areas with greater activation for Hard Subtraction than Easy Subtraction (p<.05, FDR corrected, >40 contiguous voxels). SMA = Supplementary Motor Area.
(0.03 MB DOC)

Table S4 ANOVA results of the text entry task outside the scanner.
(0.04 MB DOC)

Table S5 ANOVA results of the subtraction task outside the scanner.
(0.03 MB DOC)

Table S6 Number of scans used for the analyses of the area under the curve.
(0.03 MB DOC)

Figure S1 Response times outside the scanner.
(0.53 MB TIF)

Figure S2 Accuracy data outside the scanner.
(0.71 MB TIF)
