# Supplementary material for: The Neural Correlates of Problem States: Testing fMRI Predictions of a Computational Model of Multitasking
Source: PLoS One. 2010 Sep 23;5(9):e12966. doi: 10.1371/journal.pone.0012966 (PMC2944888; doi:10.1371/journal.pone.0012966)
Supplement: Text S1 — Exploratory fMRI Analysis. This text discusses the exploratory fMRI analysis that was performed besides the Region-Of-Interest analyses that are reported in the main text. (0.04 MB DOC) [file pone.0012966.s001.doc]

Text S1. Exploratory fMRI Analysis

An exploratory analysis was performed to identify regions that responded significantly to our experimental manipulations. The results of this analysis are reported here. All analyses were performed using the general linear model implemented in SPM5. The colored circles indicating task condition (see the Method – Procedure section, first paragraph), the ‘real’ trial, and the feedback presentations were modeled for each condition separately. Realignment parameters were included as covariates and a high-pass filter with a 128 sec cutoff was applied. For each voxel, the hemodynamic response function (HRF) and its time and dispersion derivatives were fitted. Contrast images for each condition were made for the individual participants, and entered into second level random-effect group analyses. The statistical results were thresholded using a false-discovery-rate (FDR) correction for multiple comparisons of 0.05 and more than 40 contiguous voxels. The results are summarized in Table S1 (Listening > Non-Listening), Table S2 (Hard Text Entry > Easy Text Entry), and Table S3 (Hard Subtraction > Easy Subtraction). We also tested the interaction of Subtraction Difficulty and Text Entry Difficulty, however, no brain areas survived the significance test.

Results

Table S1 lists the areas with greater activation when all three tasks had to be performed as compared to when only the subtraction and text entry tasks had to be performed, reflecting activity related to the listening task. As expected, activation was found in bilateral temporal areas and in the left inferior frontal gyrus (cf. [49]). According to for instance [50], the temporal regions perform identification processes, while the left frontal area integrates the words and sentences into a coherent whole. The temporal region overlaps with the area associated with ACT-R’s aural module (see also Figure 9).

In Table S2 areas are listed where more activation was found when the text entry task was hard as compared to when it was easy, thus when a problem state was required as compared to when it was not. The first area exists of large parts of the bilateral superior and inferior parietal lobules, including the intraparietal sulcus. This region includes the predefined problem state region, and was therefore expected to show an effect. The region is associated with attention and the integration of information (e.g., [51]), and it is therefore not surprising that it is stronger activated by the increase in task-coordination that is necessary to perform the hard text entry task. A second large activated network was found in the medial frontal cortex: the left Supplementary Motor Area (SMA), the left superior medial gyrus, extending into the left and right precentral gyri (the region extends into the left inferior frontal gyrus, which we will discuss below). This network is presumably active in response to the increase of cognitive control that is necessary for the hard text entry task in combination with the subtraction task (e.g., [52]). Furthermore, the middle and inferior frontal gyri were active bilaterally. These regions are very close to ACT-R’s declarative memory area, reflecting an increase in memory retrievals necessary for interpreting words and spelling information.

A very similar set of regions was found when we compared the hard subtraction task to the easy condition: a bilateral parietal network, a control network around the medial frontal cortex, and a memory network in the middle and inferior frontal gyri (Table S3). An increase of cognitive control and information processing requirements is even clearer for the subtraction task than for the text entry task: more difficult subtraction facts have to be retrieved and ‘borrowings’ have to be processed.
