# Supplementary material for: The Neural Correlates of Problem States: Testing fMRI Predictions of a Computational Model of Multitasking
Source: PLoS One. 2010 Sep 23;5(9):e12966. doi: 10.1371/journal.pone.0012966 (PMC2944888; doi:10.1371/journal.pone.0012966)
Supplement: Text S2 — Behavior results outside the scanner. This text discusses the results of an experiment that we ran outside the fMRI scanner. This experiment was performed to test whether differences between the behavioral results of the current fMRI experiment and a previous experiment are due to performing the experiment in the scanner and the low number of participants, or to the slightly different interface. (0.04 MB DOC) [file pone.0012966.s002.doc]

Text S2. Behavior Results outside the Scanner

Here we report the results of the experiment that we ran outside the fMRI scanner. This experiment was performed to test whether differences between the behavioral results of the current fMRI experiment and the previous experiment (Experiment 3, [7]) are due to performing the experiment in the scanner and the low number of participants, or to the slightly different interface.

Results

Participants

Twenty students of Carnegie Mellon University participated in the experiment (11 women, average age 20.6, range 18-23). All participants had normal or corrected-to-normal vision and normal hearing. Informed consent as approved by the Institutional Review Boards at Carnegie Mellon University and the University of Pittsburgh was obtained before the experiment. Participants received US$ 10 for performing the experiment.

Results

Outliers in reaction times were eliminated by means of a two step procedure. First, response times faster than 250 ms and slower than 10,000 ms were removed. Then, data exceeding 3 standard deviations from the mean per condition per participant were excluded. Overall, 2.0% of the data was discarded. Figure S1 (response times) and Figure S2 (accuracy) show the results; Table S4 (text entry) and Table S5 (subtraction) list the results of the analyses. All reported F- and p-values are from repeated measure analyses of variance (ANOVAs), all error bars depict standard errors, effects were judged significant when a .05 significance level was reached, and accuracy data were transformed using an arcsine transformation before performing ANOVAs.

Figure S1, upper panels, shows the response times on the text entry task, on the left without and on the right in combination with the listening task. A response time on the text entry task was defined as the time between entering a digit in the subtraction task and entering a letter in the text entry task. The first responses of each trial were removed (per task), as they might contain ‘start-up’ effects. An ANOVA showed that only the main effect of Subtraction Difficulty was significant (see Table S4), indicating that response times increased with Subtraction Difficulty. The interaction between Subtraction Difficulty and Text Entry Difficulty also reached significance, which is due to the increased response times in the hard-hard condition, as was predicted. All other effects were not significant.

The lower panels of Figure S1 show the response times on the subtraction task. This is the time between clicking a button in the text entry task and entering a digit in the subtraction task. Again, first responses of a trial were removed. The main effects of Subtraction Difficulty and Text Entry Difficulty reached significance (see Table S5), showing an increase in response times for both effects. Response times increased even more when both tasks were hard, as shown by the significant interaction effect of Subtraction Difficulty and Text Entry Difficulty. All other effects did not reach significance.

The two top panels of Figure S2 show the accuracy on the text entry task. The main effects of Subtraction Difficulty and Text Entry Difficulty reached significance, as did the interaction effect between Subtraction Difficulty and Text Entry Difficulty: Accuracy decreased with the two main effects, and even more when both tasks were hard. The other effects did not reach significance.

The lower panels of Figure S2 show the accuracy on the subtraction task. The main effect of Subtraction Difficulty was significant; the interaction between Subtraction Difficulty and Text Entry Difficulty showed a trend towards significance. The other tests did not reach significance.

Discussion

This experiment was performed to test why two results of the fMRI experiment were slightly different from previous experiments: First, the interaction effect in the response times of the subtraction task was absent in the fMRI experiment, and second, the pattern of response times of the text entry task was different. The current experiment, with the same interface as the fMRI experiment, did find a significant interaction effect in the response times of the subtraction task, and shows a ‘normal’ pattern in the response times of the text entry task. This suggests that the small differences in the behavioral data of the fMRI experiment are due to the changed environment and the low number of participants, not to the new interface.
