# Supplementary material for: The Neural Correlates of Problem States: Testing fMRI Predictions of a Computational Model of Multitasking
Source: PLoS One. 2010 Sep 23;5(9):e12966. doi: 10.1371/journal.pone.0012966 (PMC2944888; doi:10.1371/journal.pone.0012966)
Supplement: Table S1 — Exploratory analysis results. Areas with greater activation for Subtraction, Text Entry, and Listening than Subtraction and Text Entry alone (p<.05, FDR corrected, >40 contiguous voxels). (0.03 MB DOC) [file pone.0012966.s003.doc]

Table S1. Exploratory analysis results.

| **Gray matter of peak activation** | **Size in voxels (3x3x3 mm)** | ***t*(27)** | **MNI coordinates** |
| --- | --- | --- | --- |
| R Superior Temporal Gyrus | 1575 | 19.03 | 63, -12, 0 |
| L Superior/Middle Temporal Gyrus | 2055 | 15.5 | -57, -18, 3 |
| L Inferior Frontal Gyrus | 78 | 6.24 | -54, 24, 12 |

Areas with greater activation for Subtraction, Text Entry, and Listening than Subtraction and Text Entry alone (*p < .05, FDR corrected, >40 contiguous voxels)*.
