# Supplementary material for: The Neural Correlates of Problem States: Testing fMRI Predictions of a Computational Model of Multitasking
Source: PLoS One. 2010 Sep 23;5(9):e12966. doi: 10.1371/journal.pone.0012966 (PMC2944888; doi:10.1371/journal.pone.0012966)
Supplement: Table S2 — Exploratory analysis results. Areas with greater activation for Hard Text Entry than Easy Text Entry (p<.05, FDR corrected, >40 contiguous voxels). SMA = Supplementary Motor Area. (0.03 MB DOC) [file pone.0012966.s004.doc]

Table S2. Exploratory analysis results.

| **Gray matter of peak activation** | **Size in voxels (3x3x3 mm)** | ***t*(27)** | **MNI coordinates** |
| --- | --- | --- | --- |
| L SMA / L Superior Medial Gyrus / L Inferior Frontal Gyrus | 1287 | 9.16 | -3, 6, 60 |
| R Middle Frontal Gyrus | 318 | 8.18 | 42, 36, 33 |
| L & R Superior/Inferior Parietal Lobules | 1115 | 7.49 | -9, -69, 51 |
| L Middle Frontal Gyrus | 233 | 5.94 | -42, 45, 21 |
| R Insula Lobe | 123 | 5.28 | 30, 27, 0 |

Areas with greater activation for Hard Text Entry than Easy Text Entry (*p < .05, FDR corrected, >40 contiguous voxels)*. SMA = Supplementary Motor Area.
