# Supplementary material for: The Neural Correlates of Problem States: Testing fMRI Predictions of a Computational Model of Multitasking
Source: PLoS One. 2010 Sep 23;5(9):e12966. doi: 10.1371/journal.pone.0012966 (PMC2944888; doi:10.1371/journal.pone.0012966)
Supplement: Table S3 — Exploratory analysis results. Areas with greater activation for Hard Subtraction than Easy Subtraction (p<.05, FDR corrected, >40 contiguous voxels). SMA = Supplementary Motor Area. (0.03 MB DOC) [file pone.0012966.s005.doc]

Table S3. Exploratory analysis results.

| **Gray matter of peak activation** | **Size in voxels (3x3x3 mm)** | ***t*(27)** | **MNI coordinates** |
| --- | --- | --- | --- |
| R Superior Frontal Gyrus | 113 | 7.61 | 27, 12, 57 |
| R Supra Marginal Gyrus /  R Inferior Parietal Lobule | 485 | 7.58 | 48, -39, 45 |
| R Middle Frontal Gyrus | 215 | 6.69 | 45, 42, 24 |
| L Middle / Inferior Frontal Gyrus | 301 | 6.53 | -48, 36, 24 |
| L Inferior Parietal Lobule | 317 | 6.48 | -36, -48, 39 |
| L SMA | 90 | 5.50 | 3, 21, 45 |

Areas with greater activation for Hard Subtraction than Easy Subtraction (*p < .05, FDR corrected, >40 contiguous voxels)*. SMA = Supplementary Motor Area.
