# Supplementary material for: The Neural Correlates of Problem States: Testing fMRI Predictions of a Computational Model of Multitasking
Source: PLoS One. 2010 Sep 23;5(9):e12966. doi: 10.1371/journal.pone.0012966 (PMC2944888; doi:10.1371/journal.pone.0012966)
Supplement: Table S4 — ANOVA results of the text entry task outside the scanner. (0.04 MB DOC) [file pone.0012966.s006.doc]

Table S4. ANOVA results of the Text Entry Task outside the scanner.

|  | **Response Times** | | | **Accuracy** | | |
| --- | --- | --- | --- | --- | --- | --- |
| ***Source*** | ***F(1,19)*** | ***p*** | ***η_p_^2^*** | ***F(1,19)*** | ***p*** | ***η_p_^2^*** |
| Listening | 1.50 | .236 | .07 | < 1 | - | - |
| Subtraction | 69.49 | < .001 | .79 | 15.62 | < .001 | .45 |
| Text Entry | < 1 | - | - | 38.63 | < .001 | .67 |
| Listening x Subtraction | 1.62 | .219 | .08 | 2.00 | .173 | .10 |
| Listening x Text Entry | 1.37 | .256 | .07 | < 1 | - | - |
| Subtraction x Text Entry | 19.85 | < .001 | .51 | 16.03 | < .001 | .46 |
| Listening x Sub. x Text Entry | 2.57 | .126 | .12 | 3.06 | .096 | .14 |
