# Supplementary material for: The Neural Correlates of Problem States: Testing fMRI Predictions of a Computational Model of Multitasking
Source: PLoS One. 2010 Sep 23;5(9):e12966. doi: 10.1371/journal.pone.0012966 (PMC2944888; doi:10.1371/journal.pone.0012966)
Supplement: Table S5 — ANOVA results of the subtraction task outside the scanner. (0.03 MB DOC) [file pone.0012966.s007.doc]

Table S5. ANOVA results of the Subtraction Task outside the scanner.

|  | **Response Times** | | | **Accuracy** | | |
| --- | --- | --- | --- | --- | --- | --- |
| ***Source*** | ***F(1,19)*** | ***p*** | ***η_p_^2^*** | ***F(1,19)*** | ***p*** | ***η_p_^2^*** |
| Listening | < 1 | - | - | < 1 | - | - |
| Subtraction | 139.23 | < .001 | .88 | 52.86 | < .001 | .74 |
| Text Entry | 31.99 | < .001 | .63 | 6.10 | .023 | .24 |
| Listening x Subtraction | < 1 | - | - | < 1 | - | - |
| Listening x Text Entry | < 1 | - | - | 1.93 | .181 | .09 |
| Subtraction x Text Entry | 22.09 | < .001 | .54 | 4.19 | .055 | .18 |
| Listening x Sub. x Text Entry | < 1 | - | - | 1.26 | .276 | .06 |
