# Supplementary material for: The Neural Correlates of Problem States: Testing fMRI Predictions of a Computational Model of Multitasking
Source: PLoS One. 2010 Sep 23;5(9):e12966. doi: 10.1371/journal.pone.0012966 (PMC2944888; doi:10.1371/journal.pone.0012966)
Supplement: Table S6 — Number of scans used for the analyses of the area under the curve. (0.03 MB DOC) [file pone.0012966.s008.doc]

Table S6. Number of scans that was taken into account for the analyses of the area under the curve, per condition.

| **Subtraction** | **Text Entry** | **Listening** | **Scans** |
| --- | --- | --- | --- |
| Easy | Easy | No | 23 |
| Easy | Hard | No | 23 |
| Hard | Easy | No | 31 |
| Hard | Hard | No | 33 |
| Easy | Easy | Yes | 23 |
| Easy | Hard | Yes | 24 |
| Hard | Easy | Yes | 32 |
| Hard | Hard | Yes | 34 |
