# Supplementary figures and images for: The Neural Correlates of Problem States: Testing fMRI Predictions of a Computational Model of Multitasking
Source: PLoS One. 2010 Sep 23;5(9):e12966. doi: 10.1371/journal.pone.0012966 (PMC2944888; doi:10.1371/journal.pone.0012966)

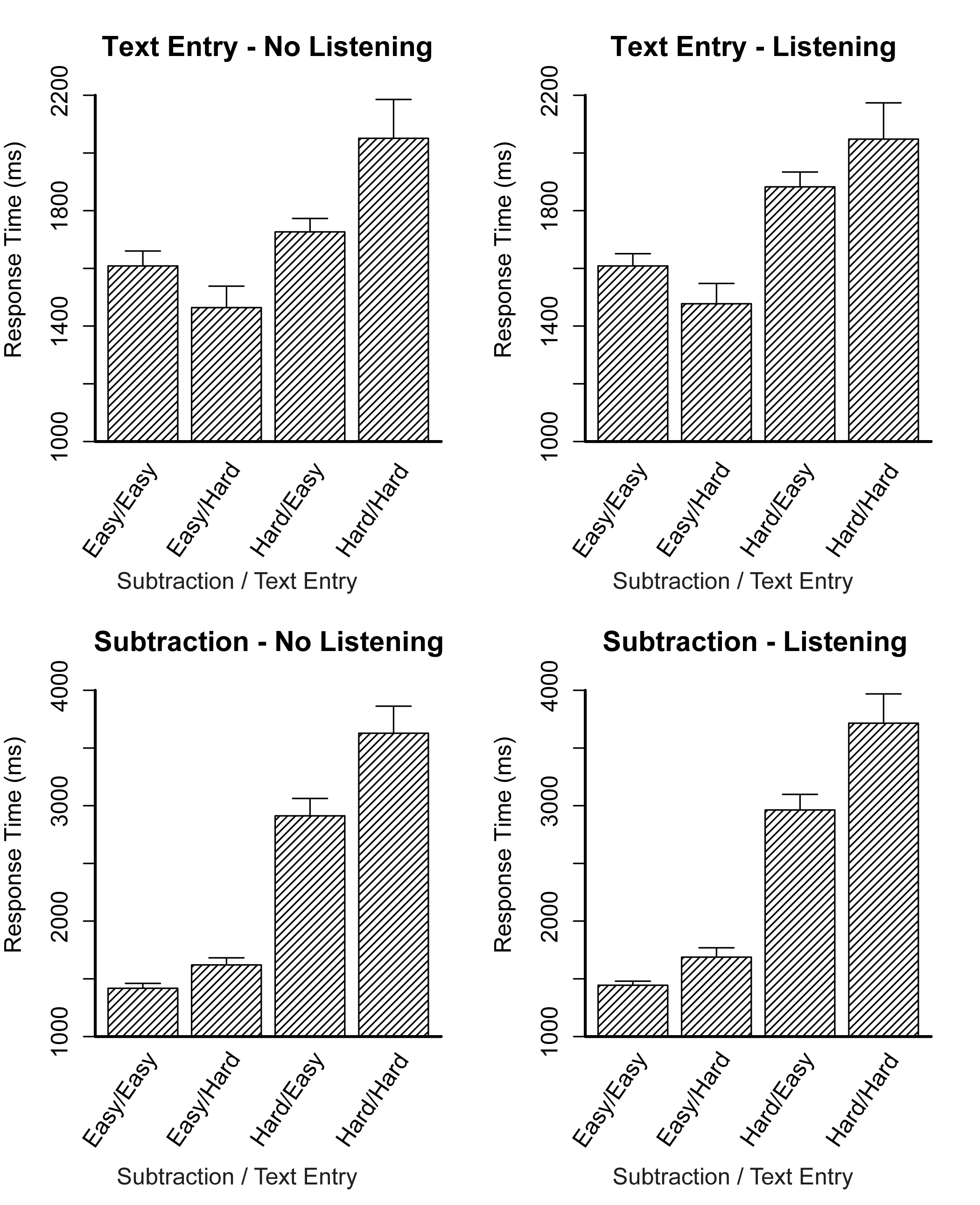

Supplement: Figure S1 — Response times outside the scanner. (0.53 MB TIF) [file pone.0012966.s009.tif]

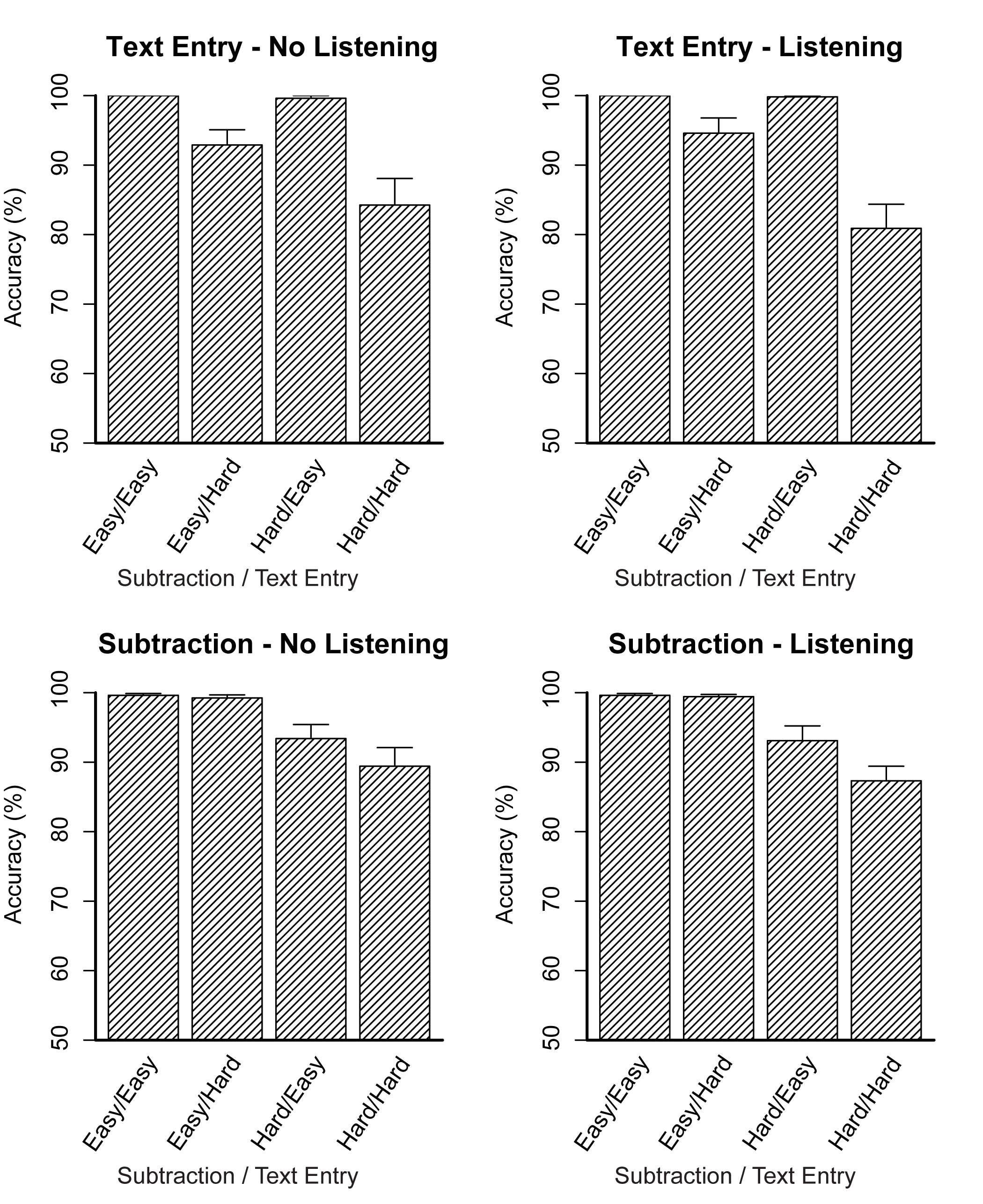

Supplement: Figure S2 — Accuracy data outside the scanner. (0.71 MB TIF) [file pone.0012966.s010.tif]
